# Classifying fossil Darwin wasps (Hymenoptera: Ichneumonidae) with geometric morphometrics of fore wings

**DOI:** 10.1371/journal.pone.0275570

**Published:** 2022-11-17

**Authors:** Alexandra Viertler, Hannes Baur, Tamara Spasojevic, Bastien Mennecart, Seraina Klopfstein

**Affiliations:** 1 Institute of Ecology and Evolution, University of Bern, Bern, Switzerland; 2 Life Sciences, Natural History Museum Basel, Basel, Switzerland; 3 Department of Invertebrates, Natural History Museum Bern, Bern, Switzerland; Laboratoire de Biologie du Développement de Villefranche-sur-Mer, FRANCE

## Abstract

Linking fossil species to the extant diversity is often a difficult task, and the correct interpretation of character evidence is crucial for assessing their taxonomic placement. Here, we make use of geometric morphometrics of fore wings to help classify five fossil Darwin wasps from the Early Eocene Fur Formation in Denmark into subfamilies and often tribes. We compile a reference dataset with 342 fore wings of nine extant subfamilies and nine relevant fossil species. Since geometric morphometrics was mostly ignored in the past in Darwin wasp classification, the dataset is first used to examine differences and similarities in wing venation among subfamilies. In a next step, we used the reference dataset to inform the classification of the fossil species, which resulted in the description of one new genus and five new species, *Crusopimpla weltii* sp. nov., *Ebriosa flava* gen. et sp. nov., *Entypoma*? *duergari* sp. nov., *Lathrolestes*? *zlatorog* sp. nov., and *Triclistus bibori* sp. nov., in four different subfamilies. Carefully assessing data quality, we show that the fore wing venation of fossil Darwin wasps is surprisingly suitable to assign them to a subfamily or even lower taxonomic level, especially when used in conjunction with characters from other parts of the body to narrow down a candidate set of potential subfamilies and tribes. Our results not only demonstrate a fast and useful approach to inform fossil classification but provide a basis for future investigations into evolutionary changes in fore wings of ichneumonids. The high informativeness of wing venation for classification furthermore could be harvested for phylogenetic analyses, which are otherwise often hampered by homoplasy in this parasitoid wasp family.

## Introduction

To make use of fossils, it is important to understand their affinity to their extant relatives, which is often a difficult but a necessary step to reconstruct past diversity patterns, their change over time, and to identify the processes shaping them. While molecular data has become the main source of information to reconstruct relationships among extant taxa, ancient DNA of fossils is limited to an age of about one million years [1, 2]. In older fossils, their morphology is often the only information that remains.

Landmark-based geometric morphometrics is a fast and powerful method to extract morphological information and evaluate biological shapes, shape variation, and size, and it is often useful for identifying and classifying taxa. In extant insects, geometric morphometrics of wings was more than once proven a robust method to distinguish and discriminate taxa, mostly at population, species, or genus levels, for instance in Coleoptera [3], different Diptera families [4–6] (e.g. Tabanidae, Calliphoridae, Culicidae), Hemiptera [7] (e.g. Psyllidae), and Hymenoptera [8, 9] (e.g. Apidae). However, it was less extensively explored at higher taxonomic levels, not to mention in fossils. Since in both amber and compression fossils, the wings are often relatively flat and especially well preserved, geometric morphometrics offers an important and straight-forward approach to quantify relatedness of fossil insect species to extant taxa. And although it is a not yet widely used method, it has already been proven efficient in some fossil hymenopteran groups, mainly bees [10–13], ants [14], social wasps [15], but also parasitoid wasps [16].

We here apply geometric morphometrics on fore wings of Darwin wasps to inform about their taxonomic placements. The Darwin wasps (Hymenoptera: Ichneumonidae) is the largest wasp family, with over 25’000 described extant species [17]. To date, there are 303 fossil species of Darwin wasps described, ranging in age from a few Cretaceous fossils, to the rather abundant fossil record of the Late Eocene, and finally to the much younger ones of the Miocene and Pliocene [18, 19]. Although it is relatively easy to distinguish an ichneumonid from other wasp families, the large number of subfamilies in Ichneumonidae (42 extant and 5 fossil) complicates the integration of new fossil taxa into the extant classification. Most ichneumonid subfamilies have been shown to be monophyletic in molecular studies [20, 21], but a subfamily placement based on morphology alone is often hard, even for some extant species, since many characters are highly homoplastic [22].

Ichneumonids have a highly conserved wing venation, which is maybe the reason why their fore wings were used sporadically before to evaluate taxonomic affiliations with morphometric analyses. In extant species for instance, it was applied to confirm species-groups [23], in fossils to confirm taxonomic positions among the oldest, extinct subfamilies [24], and also more recently to support the taxonomic placement of a fossil, where affiliation was uncertain between two subfamilies [25]. Furthermore, a handful of subfamilies are regularly keyed out by specific, unique characters in their fore wing [21], and several wing characters have also proven useful in identification keys at lower taxonomic levels [20, 21]. Despite their high species number, geometric morphometrics in wings of Darwin wasps was never applied to a larger extent, and only included a few species so far.

For ichneumonids, the second oldest excavated formation site after the K-Pg boundary is the Fur Formation in Denmark from the Ypresian epoch (56–54.5 mya), a marine Lagerstätte with a great abundance of insect fossils [26, 27]. It is assumed that the insect fossils originated from paratropical woodlands near the former southwestern Scandinavian coast [26, 28], and it remains somewhat unclear why such an abundance of them was found in these marine sediments, but transport via storms and also long-distance migration of females were discussed [29]. For a long time, *Pimpla stigmatica* Henriksen 1922 was the only described fossil ichneumonid from this formation. Recently, several new ichneumonids were described from Fur [25, 30] and now, including *Pimpla stigmatica*, there are eleven fossil species known from this formation. Rust [26] included 110 specimens of ichneumonids from the Fur Formation in his study of the biostratinomy of insects in this Lagerstätte, which highlights the high number of these wasps at the locality. He discussed different influences and aspects of how the insects fossilised in general and highlights two colour morphs in ichneumonids as well as two ways in which they lie in the sediments. The reasons for the different position in the embedment is not well understood, as are many other taphonomic aspects. When fossil wasps are embedded in compressed sediments, it might be difficult to identify certain structures, due to decay and deformation during the fossilisation process [31, 32]. Therefore, it is most likely one ends up with several possible subfamilies to place a fossil species in, since key characters are usually indiscernible in fossils.

We here compile an extensive reference dataset of fore wings of 342 species from nine subfamilies, including nine previously described fossil species from the Fur Formation [30], chosen to match the candidate subfamilies for the placement of five new studied Fur Formation fossils, in a similarity-based geometric morphometric approach. With this dataset, we aimed to answer the following questions: i) How well can the extant subfamilies be evaluated from one another? ii) With which extant subfamilies can fossil specimens be affiliated and how well supported is this classification? iii) Is it possible to make fossil assignments at a lower taxonomic level (tribe or genus)?

## Materials and methods

### Fossil specimens and morphological analysis

The fossil material described here was collected by Erwin Rettig and Jan Verkleij and is stored in the Fur Museum in Nederby (FUR). No permits were required for the described study, which complied with all relevant regulations. Photos of the newly described fossils were made with a Keyence VHX 600 camera system with a magnification of 50–200 under ethanol (to enhance the contrast), using stacking techniques. For each fossil, an interpretative illustration was made in Adobe Photoshop (v. 21.2.3), using both part and counterpart of the fossil and several photos of details as templates. The interpretative illustrations are coloured in different grey tones according to the visible colouration of the corresponding body parts. Solid lines indicate certain interpretation, whereas dotted lines illustrate uncertain interpretations.

**Fig 1:**
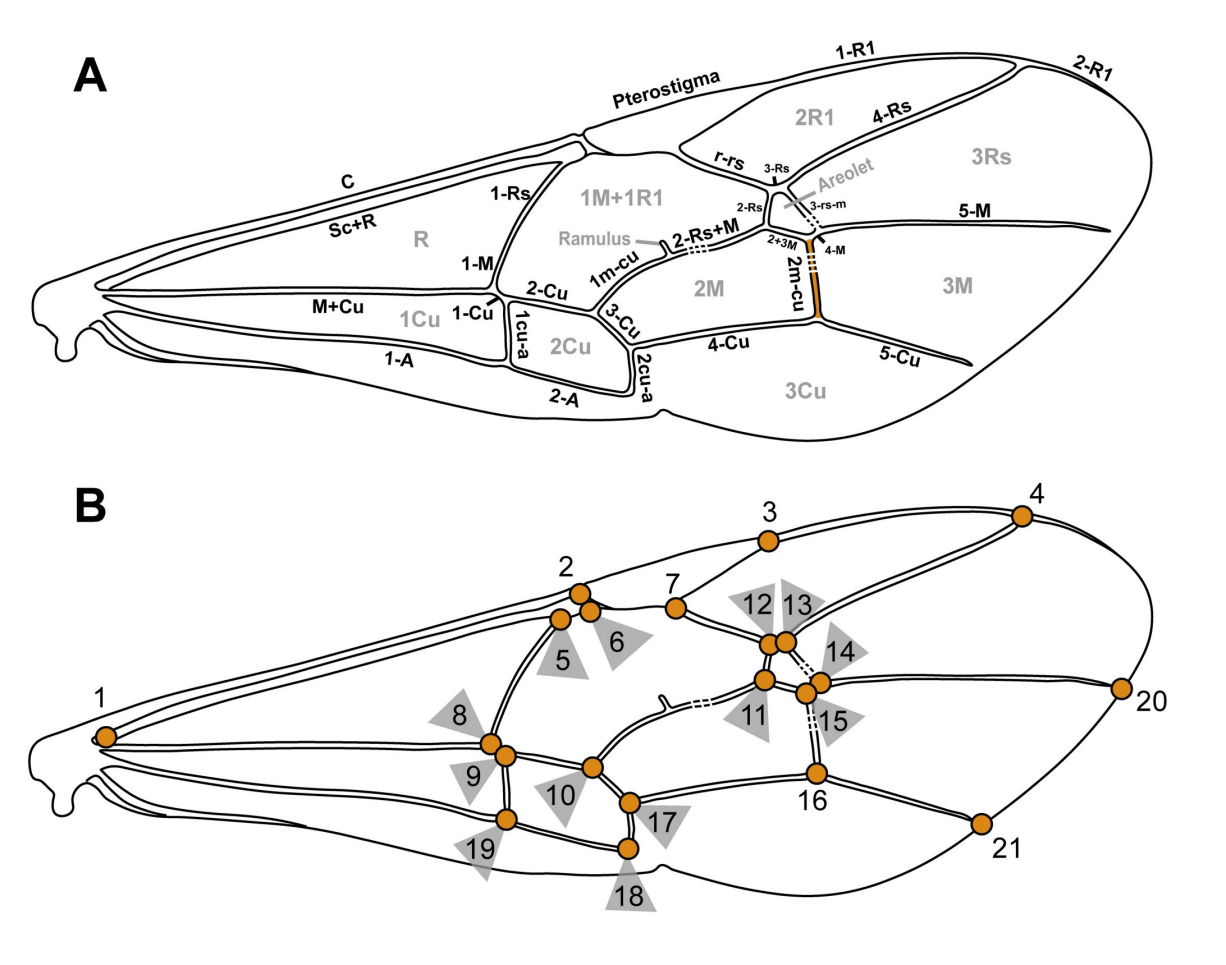
Fore wing venation shape and landmark placement. (A) Wing vein terminology used throughout the study, shown on an idealised ichneumonid fore wing. Vein 2m-cu marked orange; on this vein, a curve with six semilandmarks was added. (B) Placement of 21 fixed landmarks.

The morphological terminology follows Broad et al. [21], except for the wing terminology, which is illustrated in Fig 1A for the fore wing and follows Spasojevic et al. [33] for the hind wing. Tergites and sternites are numbered in the text and abbreviated as “T1”, “T2”, etc. and “S1”, “S2” etc., respectively. Measurements for the descriptions were made in ImageJ [34]. If paratypes are available, the range of the measurements is given, and the measurement of the holotype is provided in brackets. If both the left and right version of an appendix were complete, as it often occurred in fore or hind wings, then the average over both is given in the description. In crooked fossil specimens, the body length was measured in segments. We made use of open nomenclature [35], which is often used in palaeontology, to communicate uncertainties in the taxonomic placements. A question mark is added, in our case after the subfamily or the genus, when the identification was uncertain because a key character of this group was indiscernible.

### Taxon sampling for reference dataset

We built a large reference dataset out of extant and fossil specimens and used different subsets of it depending on the fossil species and the analysis (Table 1). For the evaluation of differences and similarities in fore wings among extant subfamilies (question i), we used 333 standardised illustrations from Townes [36–38]. This work comprises genus-level revisions of all ichneumonid subfamilies except Ichneumoninae and Hybrizontinae, with at least one habitus plate per genus, in which the wings are displayed in a standardised fashion. However, it does not include size information for the particular species in the illustrations. We thus retrieved wing or body lengths of the illustrated species from the literature using the Taxapad database [17] and used the mean if reported lengths varied within species (see S1 Table for specimen information). Townes illustrations [36–38] mainly depict female specimens, with very few exceptions. From the 42 extant subfamilies, we strategically chose nine as follows. Ctenopelmatinae, Metopiinae, Orthocentrinae, Pimplinae and Tryphoninae were included because they resemble our newly described fossil specimens the most and are thus viable candidates for their placement. Campopleginae and Cremastinae were added because of their similarity in fore wing venation and close phylogenetic relationship with one of the candidate subfamilies (see below). Finally, Ophioninae and Tersilochinae were added due to the known uniqueness of their fore wing characters [21], so we can adequately represent most of the variation found in the ichneumonid fore wing. Other closely related, but small subfamilies with only a few species were not included, nor were other subfamilies with typical body characteristics that are not found in our fossils, such as a very long and slender first tergite or a strongly pentagonal areolet in the fore wing. The species included from the nine chosen subfamilies are listed in S1 Table, and included 37 Campopleginae, 23 Cremastinae, 81 Ctenopelmatinae, 28 Metopiinae, 36 Ophioninae, 22 Orthocentrinae, 52 Pimplinae, 16 Tersilochinae, and 38 Tryphoninae species.

**Table 1:** An overview of the datasets and analyses.

Question—analysis	Taxa	Removed LM
Data quality assessment 1	Campopleginae (N = 27)	Semilandmarks
Data quality assessment 2	Campopleginae (N = 14)	LM1 + Semilandmarks
Data quality assessment 3	The five new fossils (FUR #11550, FUR #11112, #11264, FUR #10651, FUR #13809)	LM 1, 2, 18, 19
**i Procrustes distances**	333 extant (nine subfamilies)	Semilandmarks
**i Shape vs. size**	333 extant (nine subfamilies)	Semilandmarks
**i CVA**	333 extant (nine subfamilies)	none
**CVA reduced**	281 extant (seven subfamilies)	none
**ii PCA with all fossils**	281 extant (seven subfamilies), nine fossils Pimplinae, five new fossils	none
**ii PCA and CVA for each fossil**	Information for each fossil below (grey)	Information for each fossil below
ii **classification** *Crusopimpla weltii* sp. nov. (holotype, FUR #11550)	Ctenopelmatinae, Pimplinae (including fossil Fur Pimplinae), Tryphoninae	LM 1 + 18
ii **classification** *Ebriosa flava* gen. et sp. nov. (holotype, FUR #11112)	Ctenopelmatinae, Tryphoninae	LM 1, 2 +18
ii **classification** *Entypoma*? *duergari* sp. nov. (holotype, FUR #11264)	Orthocentrinae, Pimplinae (including fossil Fur Pimplinae), Tryphoninae	LM 1
ii **classification** *Lathrolestes*? *zlatorog* sp. nov. (holotype, FUR #10651)	Ctenopelmatinae, Tryphoninae	LM 1 + 19
ii **classification** *Triclistus bibori* sp. nov (holotype, FUR #13809)	Metopiinae, Campopleginae, Ctenopelmatinae	lM 1, 18 + 19
ii **classification** *Triclistus bibori* sp. nov (paratype, FUR #11215)	Metopiinae, Campopleginae, Ctenopelmatinae	LM 1
ii **classification** *Triclistus bibori* sp. nov (paratype, FUR #13077)	Metopiinae, Campopleginae, Ctenopelmatinae	LM 1
**iii between-group PCA**	Information for each fossil above (grey)	Information for each fossil above (grey)

Fossil specimens with candidate subfamilies and the excluded landmarks for each of the fossil specimen and analysis (in grey). When semilandmarks were removed (LM 22–27), the curve shape of 2m-cu was excluded.

For the questions of classification of our new fossil specimens (questions ii & iii), we pre-selected candidate subfamilies based on body characteristics from the set of five potential candidates (Ctenopelmatinae, Metopiinae, Orthocentrinae, Pimplinae and Tryphoninae) (Table 1). For *Triclistus bibori* sp. nov., we ended up with a single choice (i.e., Metopiinae) based on body characteristics (for details, see taxonomic section on this species). In order to still evaluate the power of our approach for placing fossils in a subfamily, we here included two related subfamilies which have a very similar fore wing venation (Campopleginae & Ctenopelmatinae). In *Crusopimpla weltii* sp. nov. and *Entypoma*? *duergari* sp. nov., Pimplinae was among the candidate subfamilies for placement. In addition to the extant taxa, we there also added nine previously described fossil species from the Fur Formation (S1 Table), all of which belong to this subfamily [30] (in the tenth known species from Fur, the specimen’ fore wings were too distorted to be used in geometric morphometrics).

### Landmarks for geometric morphometrics

To obtain shape information of the fore wing venation, we placed 21 fixed landmarks (LM) and one curve with six semilandmarks along vein 2m-cu (Fig 1B) on each digital image using the softwares tpsUtil (v. 1.78, [39]) and tpsDig2 (v. 2.31, [40]). The semilandmarks were chosen because the shape of 2m-cu has been mentioned previously as potentially informative for ichneumonid higher classification [21, 30]. Size information was included by setting the scale manually for each specimen. Landmarks were placed on the left fore wing in each illustrated extant taxon. In each fossil, there is at least one landmark that was placed with some uncertainty due to incomplete preservation or difficulties in the interpretation of the wings (Table 1). In all fossil wing analyses and corresponding other analyses, we excluded these uncertain landmark(s) for each fossil specimen.

Despite evidence for sexual dimorphism in wings in some ichneumonids [41, 42], we did not consider it in our study. Nearly all specimens in our reference dataset were female, and in fossils, the sex can only be clearly determined if there is a visible ovipositor or well-preserved male genital. In the case of *Ebriosa flava* gen. et sp. nov., *Triclistus bibori* sp. nov., and *Lathrolestes*? *zlatorog* sp. nov., no ovipositor is visible, leading to the assumption that they are male specimens. Rust [26] observed in 83 complete ichneumonid specimens from the Fur Formation only 17 individuals without ovipositor, identifying only one of them as a male for sure. So, the possibility remains that our fossils might be female, with a short or not well-preserved ovipositor, but if sexual dimorphism is present, a bias in our inferred affiliations cannot be ruled out.

### Data quality assessment

As using geometric morphometrics to place fossils is a rather rarely used approach, we conducted three kinds of data quality assessments. In all three assessments, we tested for a potential bias using a Procrustes analysis with the function gpagen, followed by a Procrustes ANOVA with the function procD.lm from geomorph (vers 4.0.4, [43]) in RStudio (ver. 1.2.5041, [44] R ver. 4.0.2, [45]). Additionally, the shape data was projected in morphospace with a Principal Component Analysis (PCA) to visually identify any potential biases.

First, we checked for biases regarding landmark placement by two different persons with a subset of our extant dataset (Table 1), i.e., 27 genera of the subfamily Campopleginae, with all 21 landmarks placed on the illustrations in Townes [37]. Second, we examined possible bias when using standardised drawings of wings in comparison with photos, again using a Campopleginae subset (Table 1). A bias might occur because it is not entirely clear how the illustrations in Townes [36–38, 46] were produced, although it appears that the wings were removed from the specimens, flattened and glued on cardboard in the majority if not all cases (D. Wahl, curator of Townes collection at Utah State University, Logan, pers. commun.). To make sure that the Townes illustrations can be used for our purpose, we prepared the fore wings of 14 species from the illustrations for which we also had fresh material available (see S2 Table for taxon sampling list). In the PCA we included the species that were available as photo and as illustration, as well as an additional different but congeneric species for many of them.

Third, we aimed to identify potential biases due to preservation artefacts in fossils. The left and right fore wing (right ones being mirrored), and part and counterpart in each fossil were tested for significant shape variation, using the same approach as outlined above. This comparison included the five fossils studied here, although alternative taxon samplings would be conceivable, but would rely on making *a priori* decisions about their final placement. Since in each fossil, there was at least one landmark with an uncertain placement, we removed LM 1, 2, 18 and 19 in all our fossil data in this analysis.

### Examining informativeness of fore wings for subfamily classification

We wanted to evaluate to what extent the shape of fore wings is influenced by subfamily affiliation and/or size. For this, we used the 21 fixed LM of the extant species, performed a Procrustes analysis and calculated centroid sizes with the geomorph package [43]. To determine the allometric trends across subfamilies, a Procrustes ANOVA was implemented with the function procD.lm. A regression analysis based on the PC scores and the centroid sizes was then performed to test the shape and size correlation using MorphoJ [47]. Since there may be a link between the centroid size and the subfamily attribution (tested with a Procrustes ANOVA), we performed a pooled within-group regression. In the pooled analysis, the centroid size distribution within a predefined group (here the subfamilies) is centred to the mean of the centroid size of the groups (the mean centroid size value equals to 0) preserving the relative range of centroid size but deleting the impact of the group on the shape changes. A regression is then performed using the new centroid size configuration.

In a next step, we checked which subfamilies exhibit similar LM configuration of the wing venation and to what extent the fore wing shape venation varies within each subfamily. After minimising the distances between the LM of all extant species with a Procrustes analysis, the meanshape was calculated using the function mshape from the package geomorph [43] and the full Procrustes distances were calculated in a pairwise manner using the function procdist from the package shapes (vers. 1.2.5 [48]). We filtered the Procrustes distances, so we could calculate the mean Procrustes distance of each subfamily to another subfamily and compare the shape variation among them. The Procrustes variances per subfamily were calculated with morphol.disparity from geomorph [43].

We further analysed the shape variation of the extant taxa in a Canonical Variates Analysis (CVA) with the R package Morpho (vers. 2.9, [49]) to view the maximised amount of among-group variance, relative to within-group variance [50]. The CVA additionally computes a cross-validation test to analyse which subfamilies can be statistically differentiated from one another based on the LM configuration of the wing venation.

### Fossil placement

From here, we worked with the candidate subfamilies that resemble our new fossil specimens, removing the LM that were placed with uncertainty in the respective fossil (Table 1). For a first general visualisation, we plotted our complete reference dataset, together with all fossil specimens in a PCA. The PCA is then repeated for each fossil specimen and its candidate subfamily.

To evaluate the affiliation of the fossils we performed a cross-validated CVA with the candidate subfamilies of each new fossil (Table 1), but not yet including the fossil itself, to check how well the taxonomic groups can be distinguished by LM configuration of the fore wing venation alone. The CVA results in an overall classification accuracy, including Kappa statistics (to which extent the collected data correctly represents the measured variables, ranging from -1, where the data is representing the variables worse than random, to +1, which is a perfect agreement of data and measured variables [51]. This helps to estimate the certainty when a fossil is later placed within a subfamily.

In a next step, we tested the affiliation of each fossil with the function ‘classify’ from the package Morpho [49], using the result of the conducted CVAs. This allows us to use the reference dataset as a test dataset and define the fossil specimens as new input to be assigned to a predefined taxonomic group, in our case subfamilies. If the CVA resulted in a high classification accuracy of >80% (supported by a high Kappa value of >0.7) and the fossil specimen could then be assigned to a given subfamily with a high posterior probability (pp >0.9), we used this subfamily for a narrower evaluation to tribe level or to genus group with the function groupPCA from Morpho. To do a between-group PCA (bgPCA) is compatible in this situation, since a CVA is not feasible with small sample sizes [52], as we sometimes only had a few species available per tribe or genus. The function groupPCA calculates Euclidean distances between the tribes’ averages with the fossil specimen remaining in its own group. P-values are based on permutation testing of 10’000 replications. Resemblance of the fossil specimens with extant tribes is discussed by evaluating the Euclidean distances in the descriptions of the new fossil species.

## Results

### Data quality assessments

The results of the quality assessments are shown in S2 Table. In brief, we found no indication of any systematic biases in any of the three quality assessment analyses. Furthermore, the variation introduced by different persons setting the landmarks, by using drawings versus photographs of fore wings, and by fossilisation artefacts was in nearly all cases smaller than the variation between genera, suggesting that the method is suitable for higher-level classification.

### Landmark placing by two persons

**Fig 2:**
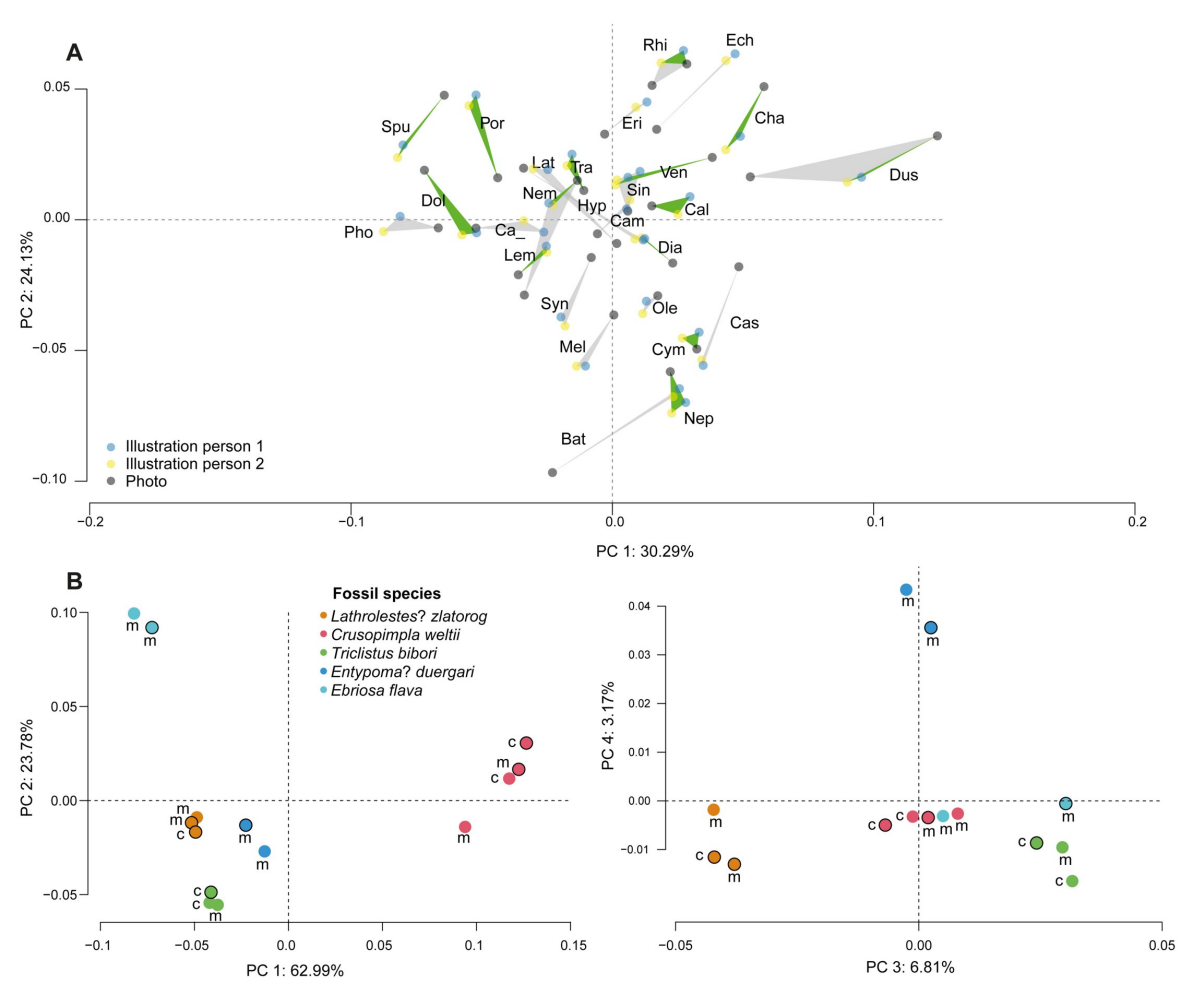
Data quality assessments. (A) PCA plot with landmarks placed by two different persons and comparison of illustrations and photos. Green triangles show the data of the same species three times, twice from an illustration, measured by two different persons, and once from a photo. Grey triangles represent species from the same genus, one species measured by two persons from an illustration and a photo of a different species, in order to additionally show some of the intra-generic variation. In those cases where our taxon sampling allowed it (*Dusona*, *Rhimphoctona*, etc.), both aspects were combined. The genus ID in the figure is composed of the first three letters of the genus (Dia–*Diadegma*) and the abbreviations, as well as the included taxa are listed in S2 Table. (B) Data quality assessment in fossils. PCA showing shape variation within and among the five newly described fossil species, “m” is representing the part (main part) and “c” the counterpart of the fossils, whereas the black outline is marking the right fore wings, the ones without black outlines the left fore wings.

Our assessment showed that the placement of landmarks on fore wings by two different persons is highly consistent, assuming they followed the same rules of placement. The variation in shape explained by different persons is very low and there seems to be no significant difference between them (R^2^: 0.8%, p = 0.8), according to a Procrustes ANOVA analysis (S2 Table). Indeed, the two replicates based on the same illustration, but with landmarks placed by different people, cluster very closely together in all cases on a PCA plot (Fig 2A, blue and yellow dot within each green triangle).

### Photos versus illustrations

When comparing photos (black dots, Fig 2A) with illustrations (blue and yellow dots, Fig 2A) of the same species (green triangles, Fig 2A), the shape data was very similar within species, and was nearly always smaller compared to the distances between genera (Fig 2A, S1 File). The variation of shape is only little explained by the use of either photo or illustration (R^2^: 9%, p = 0.5), but mostly by species (R^2^: 88.2%, p = 0.5) (S2 Table). And even when different species within a genus were sampled (grey triangles, Fig 2A), they were usually clustering closely together compared to the overall shape variation within the subfamily. Only some of the largest genera of the subfamily, such as *Dusona* Cameron, 1901, *Hyposoter* Förster, 1869 and *Bathyplectes* Förster, 1869, exhibited a rather larger distance among different species, which at times exceeded that between some of the smaller genera. These genera are known for their rather high intrageneric diversity and might in fact turn out as not monophyletic in the future [53]. The similarity of the fore wing venation shape pointed out that many species could be correctly put into their genus, no matter if a photo was used or an illustration.

### Fossilisation artefacts

Fossil specimens can show various artefacts, especially distortions resulting from the fossilisation process, which might not always be obvious but could nevertheless hamper geometric morphometric analyses. In our assessment with the fossil fore wings, we found the variation within specimen explained by side (left and right fore wing, R^2^: 0.8%, p = 0.25) and part (part and counterpart, R^2^: 0.5%, p = 0.57) rather minor: Left and right, as well as part and counterpart of the fore wings do not differ much within each fossil specimen (Fig 2B, S2 Table). We thus decided to continue with only the best-preserved imprint of the fore wing in the fossil specimens.

### Allometry in Darwin wasp wings

**Fig 3:**
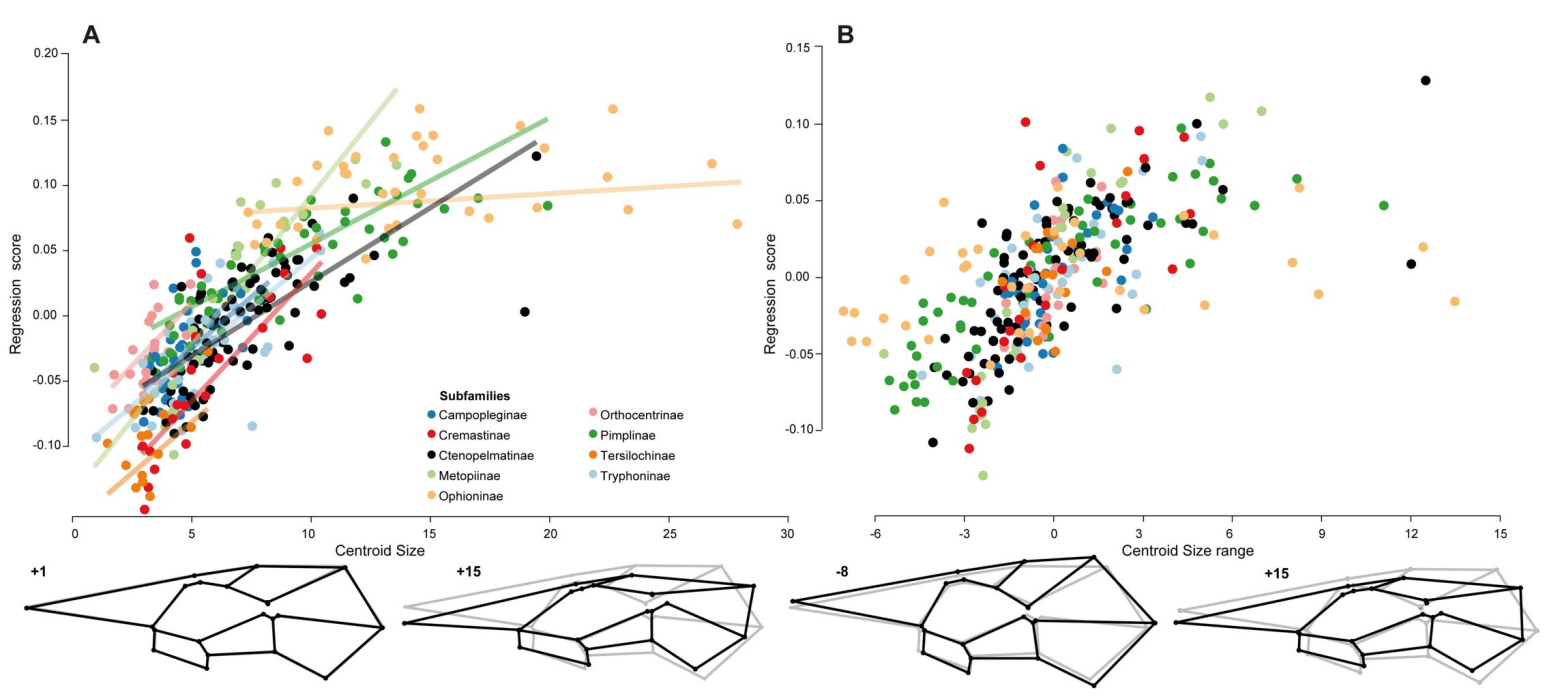
Correlation of the centroid size and the wing shape in ichneumonid subfamilies. (A) Regression analysis based on the raw data, where centroid size is plotted against the regression score, with linear regressions slopes shown by subfamily. (B) Centroid size against regression score pooled by subfamilies, with the centroid size distribution of each subfamily centred with its mean on zero. The black wings at the bottom of each plot represent shape at low and high centroid size and centroid size range, respectively. The underlying grey wing represents the mean shape of all taxa.

The analysis of shape changes in wing venation related to size shows extremely significant correlation and thus strong signal for allometry (ANOVA: 21.9%, p < 0.001; regression: 21.9% predicted, p < 0.001, Table 2 & S4 Table). While small wings possess an enlarged pterostigma and a generally broader fore wing, larger ones have a slender pterostigma and are narrower in the anterior-posterior axis, and stand out in having the LM 11 and LM 15 very close together, and the 2cu-a longer than 3Cu (Fig 3A). Nevertheless, it is worth noting that the centroid size within the nine different subfamilies is not random. Tersilochinae and Orthocentrinae are the smallest studied ichneumonids here, while the Ophioninae are the largest ones. Indeed, shape changes associated with both, centroid size and subfamily affiliation, are extremely significant (ANOVA: 6.6%, p < 0.001). Moreover, the allometric trajectories are not similar across subfamilies (Fig 3). Then working on residuals of the regression of this analysis would delete shape information related to phylogeny. So it is necessary to pool the data by subfamily. When pooling the data by subfamily, shape changes in wing venation related to size showed an extremely significant correlation with a strong signal for allometry (Regression: 12.1% predicted, p < 0.001, S4 Table). Thus 9.8% of the shape changes may be related to both the subfamilies and the centroid size when considering the results of the regression. Relatively smaller wings are here characterised by broader cells, especially the cells 2R1 and 3Rs, while the larger ones are narrower, causing the cells 2R1, 1M+1R1, 2M and 2Cu to be elongated (Fig 3B). It is interesting to note that the shape differences in the maximum extreme shape between the unpooled and pooled analysis (Fig 3) are related to subfamily affiliation. Indeed, the characters observed in the unpooled analysis, like the 2m-cu being very close to vein 2-Rs+M, the vein 3Cu being shorter than vein 2cu-a, and the very long vein 1-R1 that almost reaches the height of LM 20, are absent or reduced from the pooled one and are typical for the subfamily Ophioninae.

**Table 2:** Result of the procrustes ANOVA of shape with centroid size and subfamily affiliation.

	DF	SS	MS	R^2^	F	Pr(<F)
**Csize**	1	0.8234	0.82339	0.21936	151.2830	0.001
**Subfamily**	8	0.9684	0.12106	0.25800	22.2419	0.001
**Csize:Subfamily**	8	0.2474	0.03092	0.06590	5.6811	0.001
**Residuals**	315	1.7145	0.00544	0.45674		
**Total**	332	3.7537				

Abbreviations: DF: degree of freedom, SS: sum of squares, MS: mean sum of squares, Z: effect sizes, analysis was done using 1000 permutations.

### Shape variation among and within subfamilies

**Fig 4:**
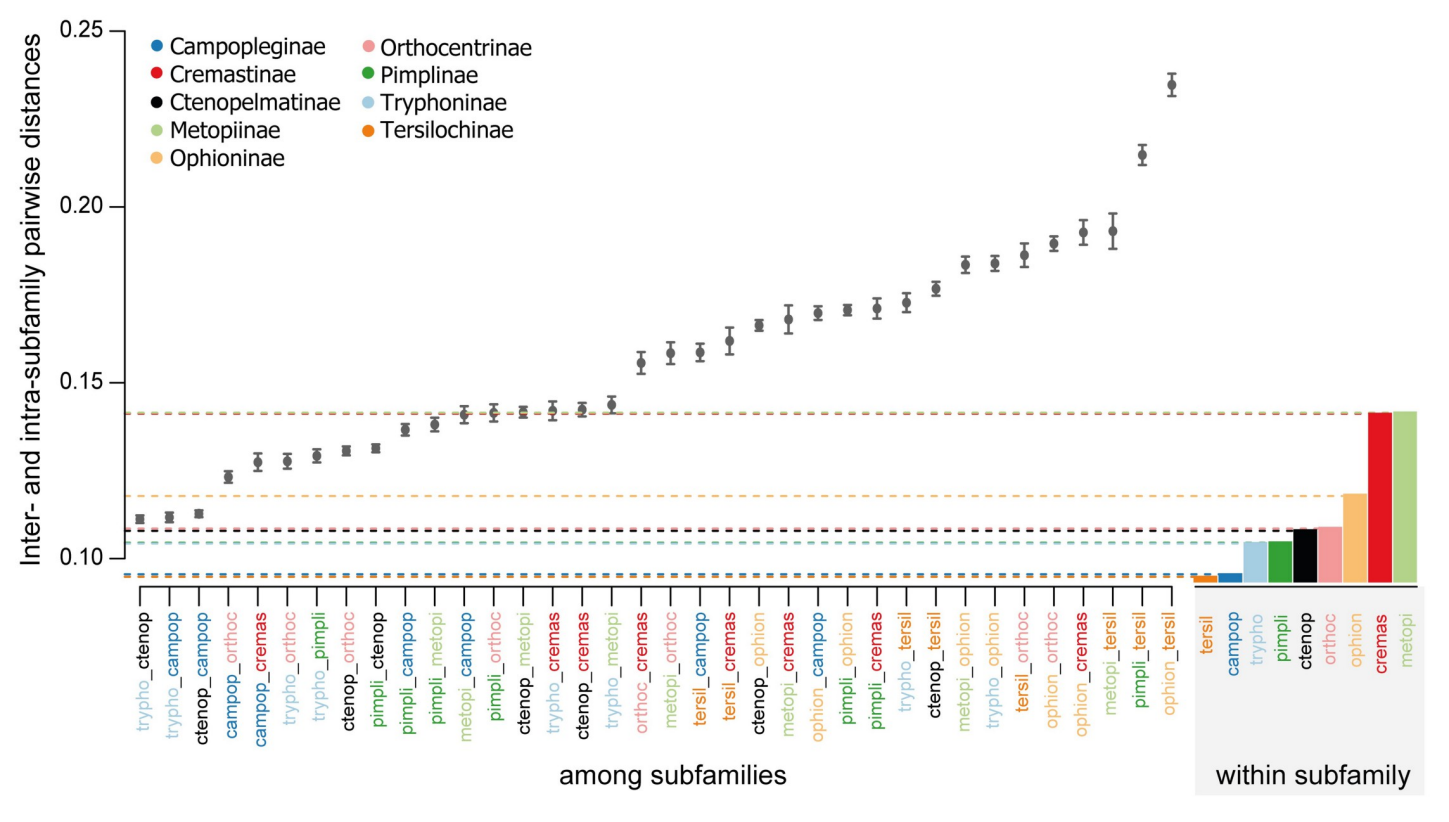
Means of Procrustes distances between species pairs among and within subfamilies. The means of the pairwise Procrustes distances for members of each subfamily pair are given in the left portion of the graph, sorted in ascending order. Whiskers indicate the standard error for each subfamily pair. On the right, the mean intra-subfamily pairwise distances are shown as barplots, with dashed lines extending to the left portion of the graph to allow direct comparison of mean intra- and inter-subfamily distances. The smallest distance between subfamiles is observed between Tryphoninae and Ctenopelmatinae and is lower than the three largest observed intra-subfamilial distances (Ophioninae, Cremastinae and Metopiinae). The largest distance is the one between Ophioninae and Tersilochinae and is about twice as high as the distances within Ophioninae. Horizontal lines and abbreviations of subfamilies are coloured according to the colour code above.

When comparing the mean Procrustes distances between the subfamilies, Tryphoninae, Ctenopelmatinae, and Campopleginae are the subfamilies that have the most similar fore wing venation to one another (Fig 4). Tersilochinae show the most distinct mean wing venation compared to others and are the most distinct from Metopiinae, Pimplinae, and Ophioninae (Fig 4).

The highest mean of Procrustes distances of pairs within subfamilies is reached by Metopiinae and Cremastinae (Fig 4, horizontal lines). The mean within those two subfamilies is higher than in many among-subfamily distances, while the mean within-subfamilies is rather consistent otherwise (S3 Table). And although the mean of Procrustes distances within-subfamily is not exactly the same as Procrustes variance, the results are very similar with the highest Procrustes variance found in Metopiinae (0.01089), closely followed by Cremastinae (0.0173) (S3 Table). Lowest Procrustes variance, and therefore the most consistent fore wing shape is found in Tersilochinae (0.00445) and Campopleginae (0.00482).

### Shape variation related to subfamily

**Fig 5:**
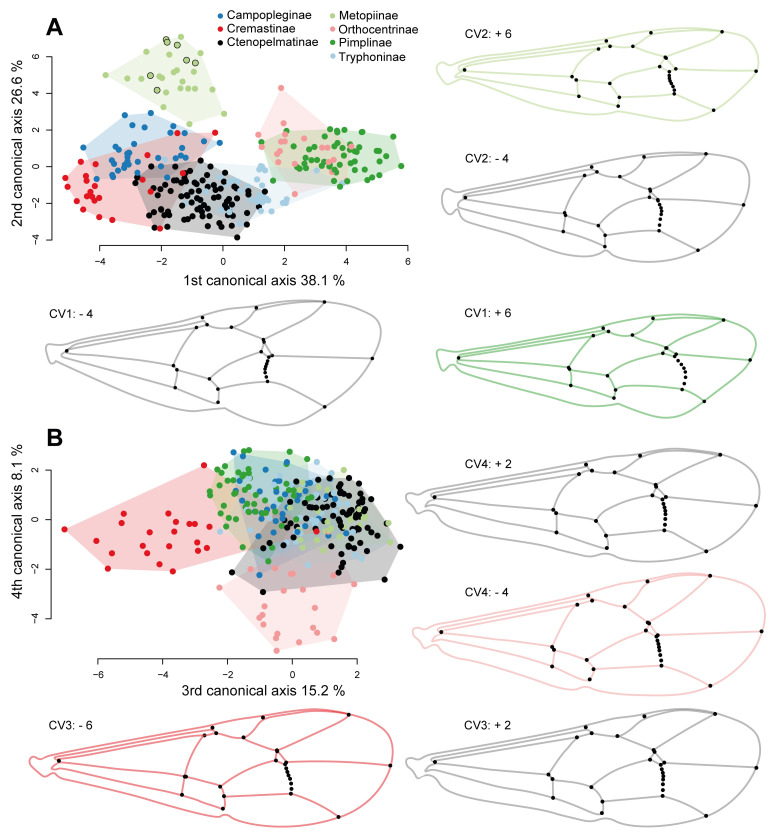
Canonical variation analysis with seven extant subfamilies. Examples of the shape at the maximum and minimum of the first four canonical axes. (A) Plot of the first two CV axes. *Metopius* Panzer, 1806 species are marked with a black contour. (B) Plot of the third and fourth CV axis. If subfamilies are more or less separate from others, we coloured the max/min shape of the wing accordingly. Grey wing outlines represent the general shape if several subfamilies overlap. The outlines on the wing illustrations are interpretative and oriented on the specimen that has the minimum/maximum CV score on the respective axis.

The shape changes accounting the subfamily affiliation are high with 25.8% (p < 0.001, Table 2). The classification results based on the pooled data and the raw data (S4 Table) are equally extremely efficient (0.3% of reclassification differences). Thus, in order to simplify the process of producing our results, we will perform our analyses only using the raw data. We conducted a cross-validated CVA of the reference taxa (Table 3A), where each individual was tested against the remaining individuals grouped in subfamilies, resulting in an overall classification accuracy of 87.9%. Ophioninae species were 100% assigned correctly, followed by over 90% correctly classified Campopleginae, Tersilochinae and Pimplinae. The subfamily Tryphoninae was the one with the fewest correct assignments. Over 20% of the Tryphoninae were falsely placed in Ctenopelmatinae, but only around 5% of Ctenopelmatinae were wrongly placed within Tryphoninae. Because about 60% (CV1 and CV2) of the shape variation split mainly Ophioninae and Tersilochinae (S1 Fig), and none of them represents a possible target subfamily of the later tested fossil specimens, we repeated the CVA without those two subfamilies (Table 3B, Fig 5).

**Table 3:** Cross-validated classification results in % from their CV scores.

**A) Subfamilies**	Campopleginae	Cremastinae	Ctenopelmatinae	Metopiinae	Ophioninae	Orthocentrinae	Pimplinae	Tersilochinae	Tryphoninae
Campopleginae	94.59	5.41	0.00	0.00	0.00	0.00	0.00	0.00	0.00
Cremastinae	0.00	82.6	8.70	0.00	4.35	4.35	0.00	0.00	0.00
Ctenopelmatinae	4.94	0.00	87.65	0.00	0.00	2.47	0.00	0.00	4.94
Metopiinae	7.14	0.00	0.00	89.29	0.00	0.00	0.00	0.00	3.57
Ophioninae	0.00	0.00	0.00	0.00	100.00	0.00	0.00	0.00	0.00
Orthocentrinae	0.00	0.00	0.00	4.55	0.00	81.82	4.55	0.00	9.09
Pimplinae	0.00	0.00	0.00	0.00	0.00	3.85	90.38	0.00	5.77
Tersilochinae	0.00	6.25	0.00	0.00	0.00	0.00	0.00	93.75	0.00
Tryphoninae	0.00	2.63	21.05	0.00	0.00	0.00	5.26	0.00	71.05
Overall classification accuracy:		87.98% (Kappa statistic: 0.86)							
**B) Subfamilies**	Campopleginae	Cremastinae	Ctenopelmatinae	Metopiinae	Orthocentrinae		Pimplinae		Tryphoninae
Campopleginae	91.89	0.00	2.70	0.00	5.41		0.00		0.00
Cremastinae	0.00	86.96	8.70	0.00	0.00		4.35		0.00
Ctenopelmatinae	3.70	2.47	85.19	0.00	2.47		0.00		6.17
Metopiinae	3.57	0.00	0.00	89.29	0.00		0.00		7.14
Orthocentrinae	0.00	0.00	0.00	4.55	72.73		13.64		9.09
Pimplinae	0.00	0.00	1.92	0.00	3.85		86.5		7.69
Tryphoninae	0.00	2.63	26.32	0.00	0.00		5.26		65.79
Overall classification accuracy:		83.3% (Kappa statistic: 0.79)							

(A) Results of CVA using nine subfamilies, including all landmarks. (B) Results of CVA using seven subfamilies, including all landmarks. The correct reclassification per subfamily is marked in grey in both analyses.

**Fig 6:**
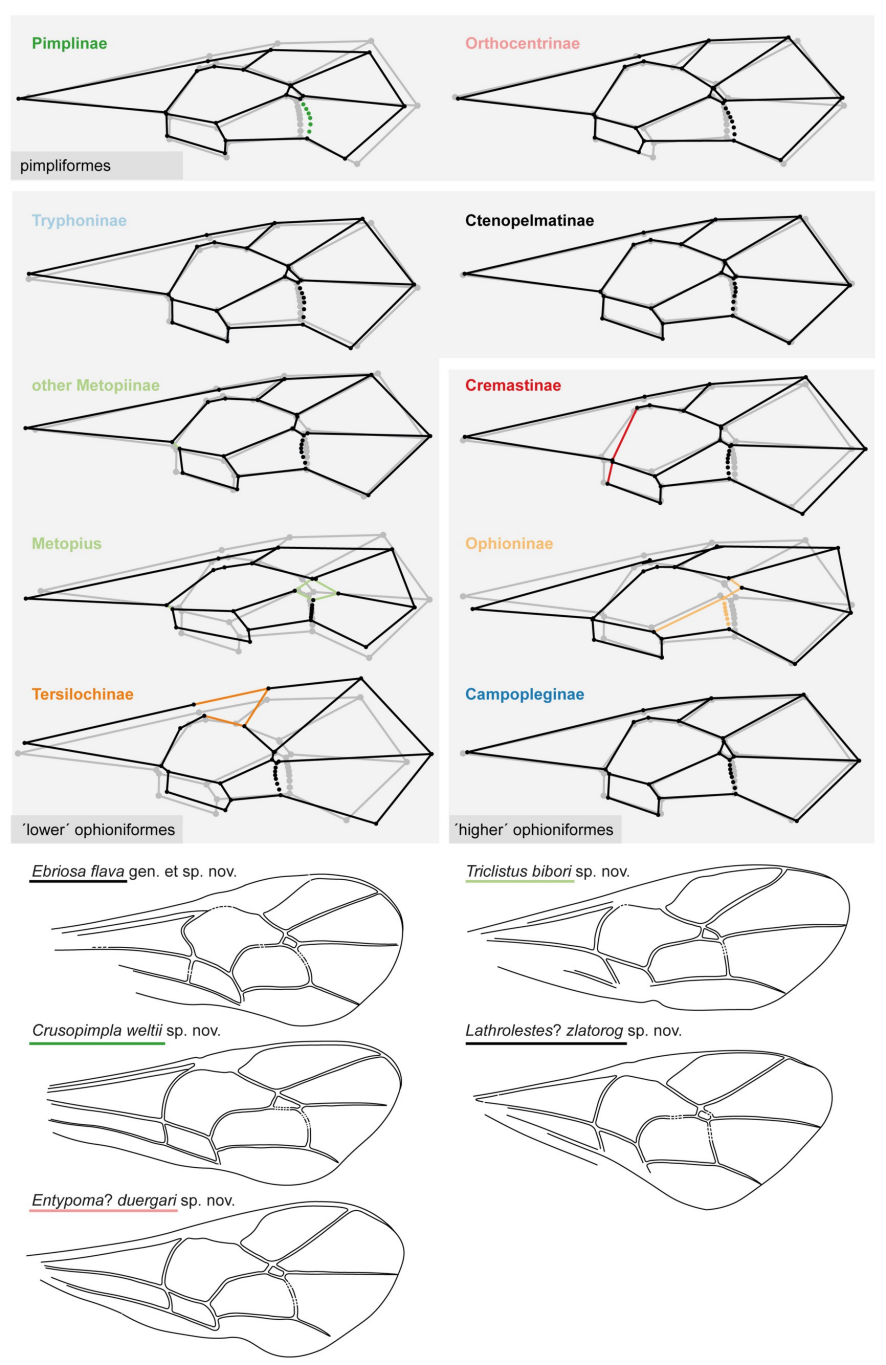
Fore wing mean shapes of subfamilies and fore wings of the five new fossil species. Mean shapes of all nine subfamilies, where Metopiinae were split into two groups: the genus *Metopius* with a very distinct fore wing venation, and the rest of Metopiinae. The mean shape of areolets are represented open (eg. Tersilochinae) or closed (eg. Campopleginae) based on the majority of included species. Group specific venation shapes are coloured in the subfamily colour code when mentioned in the results or discussion. Names of the new fossil specimens are underlined with the colour of the respective subfamily they were placed in.

Without Ophioninae and Tersilochinae, the CV1 (38.1% explained variance) separates almost all Pimplinae and Orthocentrinae, the two subfamilies of Pimpliformes in our dataset, from the other groups, which all belong to Ophioniformes. There is some overlap though with some Tryphoninae, which is probably the sister group of the other ophioniform subfamilies. Pimplinae and Orthocentrinae can be distinguished from the other subfamilies mainly by an evenly outwards-bowed 2m-cu vein and the length of vein 4Cu being longer as vein 5Cu (Fig 5A). Regarding CV1, species of Cremastinae for example represent the opposite shape of Pimplinae and Orthocentrinae, where vein 4Cu is shorter than vein 5Cu, and the 2m-cu is bowed inwards. Along CV2, 26.6% of the variation is captured, and it represents the width of the fore wing to some extent, again the curve of the 2m-cu vein, and areolet size and shape (Fig 5A). Most Metopiinae split here from the rest by having a very slender wing shape in the anterior-posterior axis, a small and slender pterostigma, narrowed cells and the 2m-cu vein slightly inward bowed and sinuous. Metopiinae also have vein 3Cu longer than vein 2cu-a and species in the genus *Metopius* possess a relatively large areolet (Fig 5). In CV3 (15.2% explained variance), all Cremastinae except *Belesica pictipennis* Tosquinet, 1896 split from the other subfamilies, mainly due to the straight and interstitial placement of vein 1cu-a to 1M + 1Rs, where 1Cu is very short or absent (also visible in the mean shape in Fig 6), and a slightly more elongate overall fore wing. In CV4, the Orthocentrinae also mostly group separately, overlapping some Ctenopelmatinae. The wing venation separates Orthocentrinae by having shorter 2R1 and 2Cu cells, a broader pterostigma and 3Cu about the same length as 2cu-a. CV5 (7.7% explained variance) separates Campopleginae from many other taxa, due to their relatively short 2Cu cell, a longer 3Cu cell, as well as the anteriorly inwards bowed 2m-cu vein (S2 Fig). CV6 (4.2% explained variance) shows a slight shape difference in some Tryphoninae that have a slightly wider fore wing, resulting in some broader 2M, 3M, and 2R1 cells (S2 Fig). Other than that, taxa in Tryphoninae seem difficult to separate entirely from taxa of Ctenopelmatinae in the CVA or the comparison of their mean shapes (Fig 6).

### Subfamily placement of the newly described fossil species

**Fig 7:**
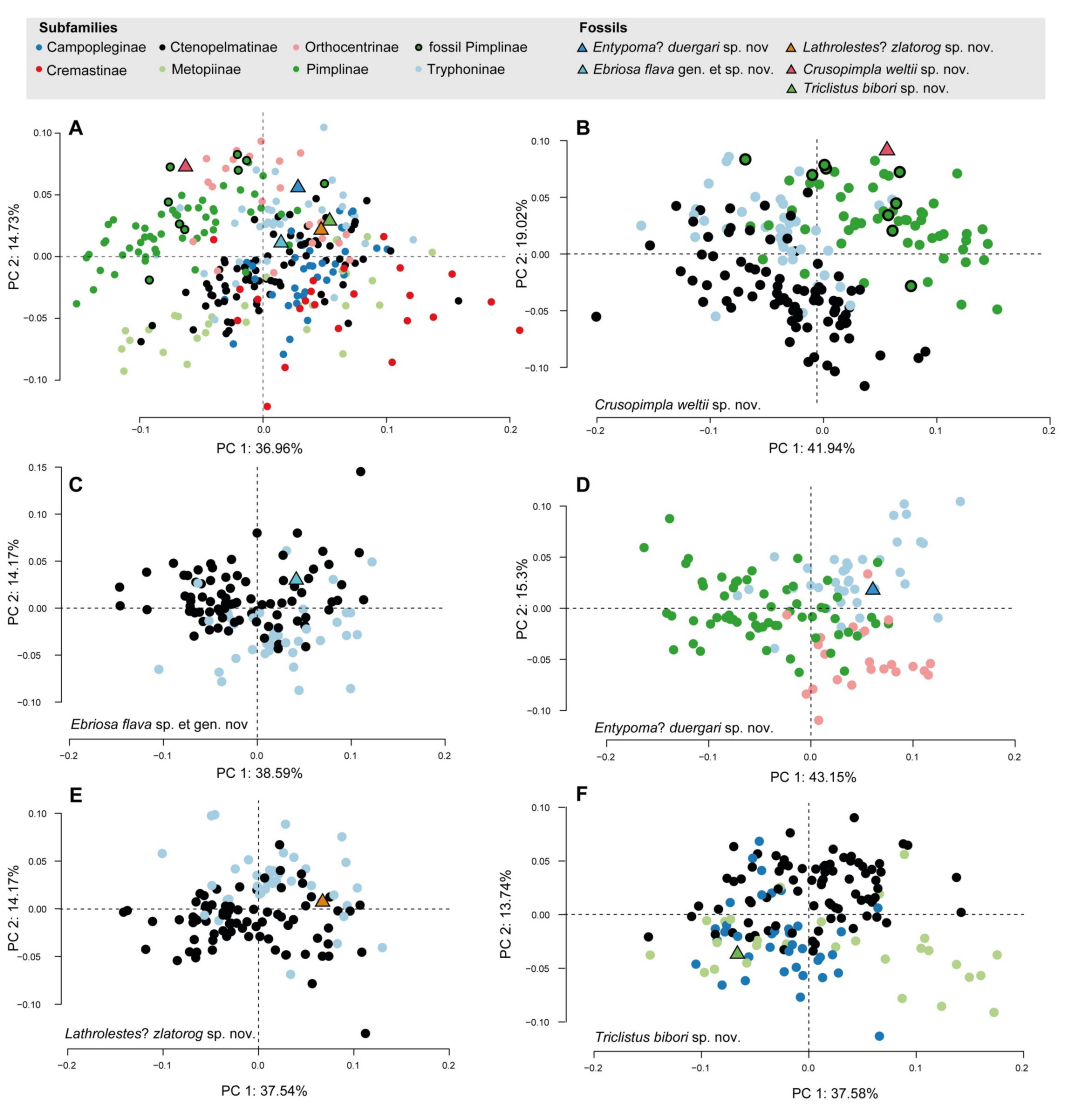
PCA analyses of fore wing shape including extant and fossil taxa. (A) PC1 against PC2 of seven subfamilies, including fossil Pimplinae and the newly described fossils. All 21 fixed and six semilandmarks were used, and the landmarks that were missing in the fossil specimens were estimated here. (B–F) PC1 and PC2 of the fossil species together with their candidate subfamilies. Landmarks of the reference subfamilies were adjusted to fit the respective fossil specimen (Table 1).

The plots of PC1 against PC2 (Fig 7) of the candidate subfamilies and the new fossil specimens show first tendencies of subfamily affiliation. Some specimens grouped better within a subfamily e.g. *Crusopimpla weltii* sp. nov. in Pimplinae (Fig 7B), while others grouped less explicitly, e.g. *Lathrolestes*? *zlatorog* sp. nov. (Fig 7E) in between Ctenopelmatinae and Tryphoninae.

The candidate subfamilies are tested in a separate CVA analysis without the fossil to see how often their members are assigned correctly in the given comparison (Table 4, light grey). Subsequently, the fossil specimen is added and assigned to an extant subfamily (Table 4, dark grey), showing the posterior probability for each candidate subfamily. In *Crusopimpla weltii* sp. nov. (FUR #11550) and *Entypoma*? *duergari* sp. nov. (FUR # 11264) the classification result of the CVA and the posterior probability of the fossil classification were considered clear enough (classification accuracy of CVA >80%, Kappa value >0.7, posterior classification >0.9), to continue the bgPCA with narrower groups of the resulting subfamily, Pimplinae in the former, Orthocentrinae in the latter case. In *Ebriosa flava* gen. et sp. nov. (FUR #11112) and *Lathrolestes*? *zlatorog* sp. nov. (FUR #10651), where the two very similarly looking subfamilies Ctenopelmatinae and Tryphoninae were considered candidate subfamilies, the classification results are less clear and for both those fossil specimens the bgPCA was conducted with tribes of both candidate subfamilies. An additional argument for testing both candidate subfamilies in *Ebriosa flava* gen. et sp. nov. (FUR #11112) is that the resulting classification disagreed with the fore wing characteristics of two bullae in vein 2m-cu, which is not caught in the geometric morphometrics analysis. The specimen of *Triclistus bibori* sp. nov. (FUR #13809), who had three uncertain and therefore removed LM (Table 1), was classified as Ctenopelmatinae (Table 4). In cell 2Cu, also represented by the here missing LM 18 and LM 19, differences are visible between the mean shapes of Ctenopelmatinae and Metopiinae (Fig 6), so we additionally included two nicely preserved paratypes in the classification analyses, where only LM 1 was uncertainly placed (Table 1). Both those paratypes (FUR #11215 and FUR #13809) were classified as Metopiinae (Table 4). For *Triclistus bibori* sp. nov. we therefore used first, tribes of all candidate subfamilies (genus *Metopius* and other Metopiinae split) and second, all genera of Metopiinae in the bgPCA, since there are no defined tribes. The resemblance of the fossil species fore wing venation, according to the Euclidean distances of the groups averages from the bgPCA, are included in the fossil species descriptions below.

**Table 4:** Cross-validated classification results and probabilities of the fossil assignment for classification.

***Crusopimpla weltii* sp. nov.**				
	Ctenopelmatinae	Pimplinae	Tryphoninae	Analysis
Ctenopelmatinae	88.8	0	11.1	CVA reference
Pimplinae	1.6	90.2	8.2	CVA reference
Tryphoninae	31.6	5.3	63.2	CVA reference
Overall classification accuracy: 83.9%, Kappa statistic: 0.746				
**Fossil Holotype *C*. *weltii* sp. nov. (FUR #11550) (posterior probability)**	<0.001	0.99	<0.001	Classification
***Ebriosa flava* gen. et sp. nov.**				
	Ctenopelmatinae	Tryphoninae		Analysis
Ctenopelmatinae	86.4	13.6		CVA reference
Tryphoninae	31.6	68.4		CVA reference
Overall classification accuracy: 80.77%, Kappa statistic: 0.55				
**Fossil Holotype *E*. *flava* gen. et sp. nov. (FUR #11112) (posterior probability)**	1	0		Classification
***Entypoma*? *duergari* sp. nov.**				
	Orthocentrinae	Pimplinae	Tryphoninae	Analysis
Orthocentrinae	72.7	22.7	4.5	CVA reference
Pimplinae	1.6	90.2	11.5	CVA reference
Tryphoninae	7.9	10.5	81.6	CVA reference
Overall classification accuracy: 84.3%, Kappa statistic: 0.74				
**Fossil Holotype *E*.? *duergari* sp. nov. (FUR #11264) (posterior probability)**	0.87	0.13	0	Classification
***Lathrolestes*? *zlatorog* sp. nov.**				
	Ctenopelmatinae	Tryphoninae		Analysis
Ctenopelmatinae	80.2	19.8		CVA reference
Tryphoninae	34.2	65.8		CVA reference
Overall classification accuracy: 75.6%, Kappa statistic: 0.45				
**Fossil Holotype *L*.? *zlatorog* sp. nov. (FUR #10651) (posterior probability)**	0.32	0.68		Classification
***Triclistus bibori* sp. nov.**				
	Campopleginae	Ctenopelmatinae	Metopiinae	Analysis
Campopleginae	91.8	8.1	0	CVA reference
Ctenopelmatinae	4.9	95	0	CVA reference
Metopiinae	7.1	3.5	89.3	CVA reference
Overall classification accuracy: 93.28%, Kappa statistic: 0.88				
**Fossil Holotype *T*. *bibori* sp. nov. (FUR #13809) (posterior probability)**	0.09	0.91	<0.001	Classification
Fossil Paratype 1 *T*. *bibori* sp. nov. (FUR #11215) (posterior probability)	0.008	0.09	0.90	Classification
Fossil Paratype 2 *T*. *bibori* sp. nov. (FUR #13077) (posterior probability)	0.007	0.001	0.99	Classification

The uncertain LM were removed for each specimen (Table 1) before the CVA was conducted. CVA classification results are in % and are coloured in light grey, classification results are coloured in dark grey.

### Systematic palaeontology

**Hymenoptera** Linnaeus, 1758

**Ichneumonidae** Latreille, 1802

**Pimplinae** Wesmael, 1844

*Crusopimpla* Kopylov, Spasojevic & Klopfstein, 2018

**Fig 8:**
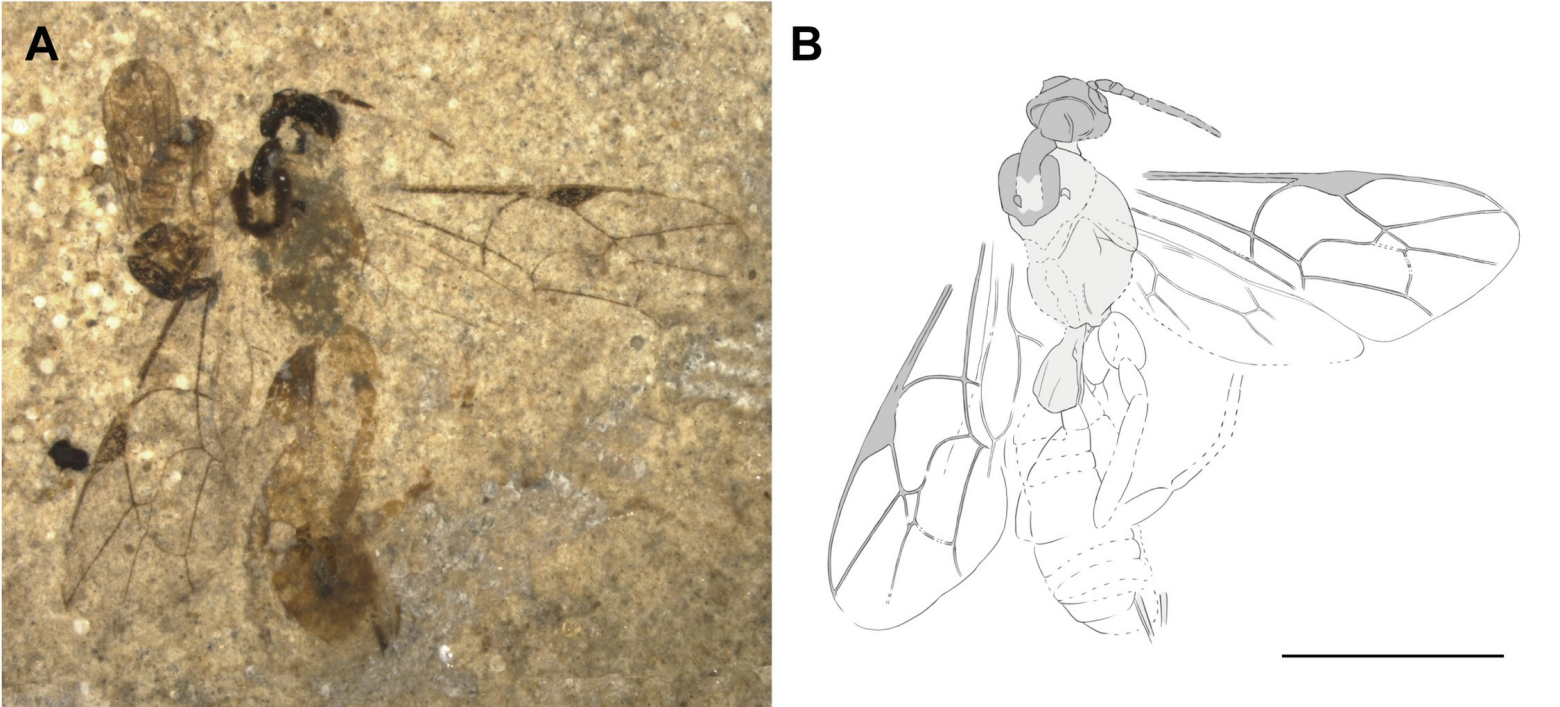
Holotype of *Crusopimpla weltii* sp. nov. (FUR #11550). (A) Photo of the fossil part. (B) Interpretative drawing, part and counterpart were used as templates. Scale bar: 2mm.

***Crusopimpla weltii*** Viertler, Spasojevic & Klopfstein, sp. nov. (Fig 8)

urn:lsid:zoobank.org:act:3F7CFB00-9C3F-4F0B-91D4-154A2D2B3612

#### Etymology

This species is named in honour of A. Viertler’s dear friend A. Welti, who is an enthusiastic scientist and shares her passion about nature in all its facets.

#### Material

Holotype: female (FUR #11550). Denmark, county: Morsø Kommune, locality: Lynghøj. Fur Formation, Ypresian epoch (56–54.5 mya). Leg: Verkleij, Jan.

#### Type preservation

Holotype in dorso-lateral view. Antenna only partially preserved. Mesosoma with several details visible. Fore- and hind wings almost complete. Hind legs partially visible. Metasoma partially preserved. Traces of female genitalia visible.

#### Systematic placement

Based on the robust body, the fore wing venation with the evenly curved 2m-cu and two bullae evenly spread on the vein, and the stout first tergite, this species resembles Pimplinae the most, but also Ctenopelmatinae and Tryphoninae cannot be ruled out entirely as possible subfamilies. The length of the ovipositor unfortunately cannot be measured, which might have helped distinguish between them.

The geometric morphometrics analysis clearly classifies the fossil as Pimplinae, placing it closest to *Crusopimpla*, followed by the tribe Ephialtini in the between-group PCA. The specimens fore wing matches the mean shape of extant Pimplinae (Fig 6) by the outwards bowed 2m-cu, the 1cu-a interstitial to 1M + 1Rs and the 4Cu longer than vein 5Cu. The Euclidean distances of the bgPCA shows that the fore wing venation of *Crusopimpla*, including *C*. *weltii* sp. nov. differs from extant tribes, but is most similar to Ephialtini.

Within Pimplinae, the vein 1Cu in the hind wing being longer than cu-a suggests *Crusopimpla* and *Scambus* Hartig, 1838, the latter belonging to Ephialtini, while the former is a genus only known from fossils and not placed in a recent tribe. *Scambus* species never have deep notauli as in this fossil and have a much shorter T1. The colouration, a darker head and mesosoma, and the orange metasoma and legs, support an affiliation to *Crusopimpla*. Nevertheless, one of the main characteristics of *Crusopimpla*, the fully carinated propodeum, is not clearly visible in this fossil, which might be an artefact of preservation.

#### Diagnosis

Seven species of *Crusopimpla* have previously been described, five of which from the Fur Formation. *C*. *weltii* sp. nov. differs from almost all other *Crusopimpla* by its orange mesosoma. In *C*. *tadushiensis* Kopylov, Spasojevic & Klopfstein, 2018 the colouration is not visible, but the clearly shorter tergites are. With the bright colouration of the mesosoma, this fossil also differs from the four fossil *Scambus* species, which have a dark brown or black mesosoma. *Crusopimpla weltii* sp. nov. combines dark antennae, fore wings with a large areolet and slender pterostigma, an interstitial 1cu-a to 1M + 1Rs, and an inclivous 1Cu vein on the hind wing, a unique combination among fossil as well as extant Pimplinae.

#### Description

Body 10.6 mm. Head, including antennae and mesoscutum dark brown or black. Rest of mesosoma bright orange. Fore wing venation and T1 brownish. Hind legs and rest of metasoma orange.

*Head*. Dimensions unclear. Antenna incomplete with only few basal segments visible; scape clearly longer than pedicel; first flagellomere about 3.7x as long as wide. Occipital carina appears dorsally complete.

*Mesosoma*. Mesoscutum with rather deep and slightly converging notauli; surface apparently smooth. Propodeum about as long as high; with complete pleural carina; traces of lateral longitudinal carina visible. Hind leg rather slender; hind coxa slightly longer than deep; hind femur about 4.6x as long as wide but might be longer; tibia shape unclear.

*Wings*. Fore wing 8.1 mm. Areolet closed, quadrate oblique with short 4M; 3rs-m similar in length to 2+3M; 2Rs half as long as 3rs-m. 2m-cu evenly curved outwards with two bullae covering about 27% of whole 2m-cu length. 4Cu 2.9x 5Cu. 4Rs almost straight. Ramulus absent. 1cu-a interstitial to 1M + 1Rs, with 1cu-a emerging at a right angle. Pterostigma 3x as long as wide. Cell 2R1 3.3x as long as wide. 5M seems tubular. 2Cu 0.8x 1M + 1Rs and about as long as r-rs. 1m-cu&2-Rs+M bowed. 3Cu about same length as 2cu-a. Hind wing with 1Cu 3x cu-a. 1Rs longer than rs-m.

*Metasoma*. Shape difficult to interpret. T1 stout, with spiracle anteriorly to middle. Latero-median carinae on more than half the length of T1, more or less parallel. Following tergites appear shorter than wide. Base of ovipositor and its sheaths present, length unclear.

**Ctenopelmatinae?** Förster, 1869

***Ebriosa*** Viertler, Spasojevic & Klopfstein, gen. nov. (feminine)

urn:lsid:zoobank.org:act:9902673E-94BD-4A09-BEB1-B2BF73F09E3D

#### Etymology

Derived from the Latin “ebriosus”, meaning drunkard. Alludes to the extremely curved M&RS vein on the fore wings, which is straight or only slightly curved in all other known ichneumonids.

#### Type species

*Ebriosa flava* gen. et sp. nov.

#### Systematic placement

The quadrate-oblique areolet and shape of T1, in combination with a dorsoventrally depressed and drop-shaped metasoma suggests Tryphoninae or Ctenopelmatinae. The two bullae in 2m-cu point to Tryphoninae, since Ctenopelmatinae often have only one, but this is not always the case. Distinct body characteristics usually used to distinguish these two subfamilies are either not visible or not preserved in this fossil: Tryphoninae have a fringe of setae on their clypeus and most female Tryphoninae bear a stalked egg on their ovipositor, whereas Ctenopelmatinae can be distinguished by a tooth on the apical margin of their fore tibia—all characters very difficult to impossible to see in compression fossils.

Comparing it with extant Tryphoninae, the rather stout body, vein 1Cu being longer than cu-a in the hind wing, the gradually widened T1, the strong latero-median carinae on T1 with the spiracle in front of the middle might point to the tribe Tryphonini, which also comprises genera with a rather large pterostigma, as in this fossil (e.g. *Ledora* Kasparyan, 1983, *Grypocentrus* Ruthe, 1855).

Within extant Ctenopelmatinae, it gets difficult to suggest further placement of this fossil, since many tribe-specific characteristics are not visible. But since Ctenopelmatinae taxa have mostly a rather slender pterostigma, many could be excluded and taxa with a wider and stouter pterostigma could be considered, such as some genera in the tribe Perilissini (e.g. *Absyrtus* Holmgren, 1859, *Tetrambon* Townes, 1970, *Nanium* Townes, 1967).

For the geometric morphometric analysis, Ctenopelmatinae and Tryphoninae were thus selected as possible subfamilies. For this fossil, three landmarks had to be removed (Table 1), which resulted in a lower classification accuracy for the reference dataset, especially for Tryphoninae (68.4% correctly assigned, Table 4). In the analysis, the fossil assigns clearly to Ctenopelmatinae with 100%, but the classification in the cross-validated CVA of Ctenopelmatinae and Tryphoninae is rather low (Table 4). This might bias the classification of the fossil. The mean shapes of both candidate subfamilies are very similar to each other (Fig 6) and the fore wing venation of the fossil specimen does not resemble any other subfamily. The between-group PCA was done with tribes from Tryphoninae and Ctenopelmatinae, where again the fossils venation does not closely resemble any of the tribes according to the Euclidean distances of the tribes averages (S5 Table). Nevertheless, the tribes with the smallest distance to the fossil are Perilissini and Pionini in Ctenopelmatinae and Oedemopsini in Tryphoninae.

The classification result suggests a placement in Ctenopelmatinae. But because of the low classification success of both subfamilies, the two bullae in the 2m-cu which are more rare in Ctenopelmatinae than in Tryphoninae, and the lack of additional informative characteristics, we place this fossil in Ctenopelmatinae as tentative. Furthermore, none of the extant genera in Tryphoninae and Ctenopelmatinae show a conspicuously sinuous M&Rs vein as seen in this fossil, and therefore we define the new genus *Ebriosa*.

#### Diagnosis

The genus differs from other Tryphoninae and Ctenopelmatinae genera in having a strongly curved M&Rs vein in the fore wing, a character not present either in any other ichneumonids. In addition, the genus is characterised by a long and wide pterostigma, a 1R1 vein that is shorter than the pterostigma length, an evenly bowed 2m-cu with 2 bullae, an at least partly areolated propodeum, and a broad and gradually widening T1 with the spiracle in the anterior half and latero-median carinae reaching beyond the middle.

**Fig 9:**
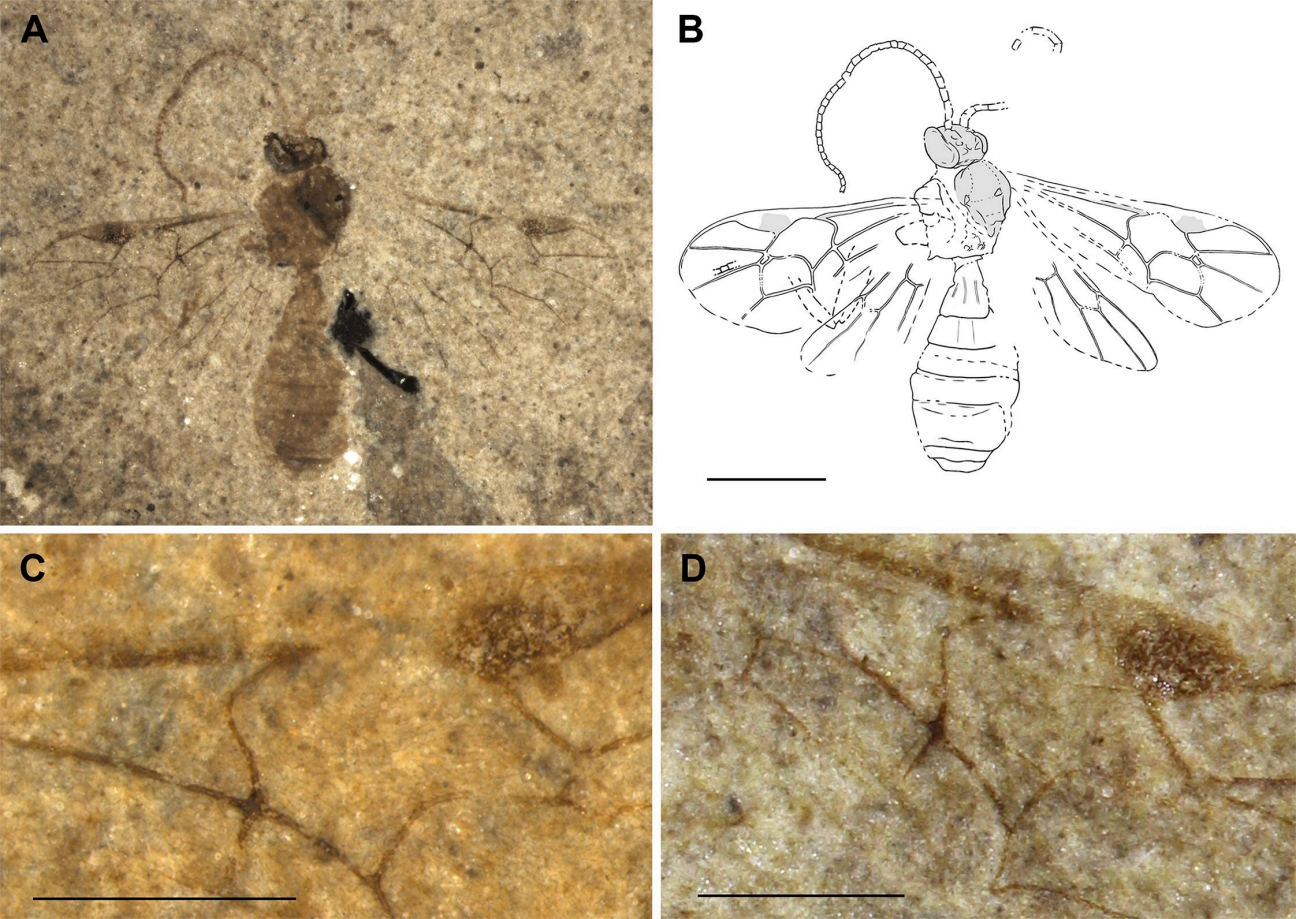
Holotype of *Ebriosa flava* gen. et sp. nov. (FUR #11112). (A) Photo of the fossil part. (B) Interpretative drawing, fossil part and counterpart were used as templates. (C) Strongly bowed M&Rs vein of the left fore wing, which is the name giving character. (D) Fore wing of the paratype #13812. Scale bars: (B) 2mm, (C) 1mm.

***Ebriosa flava*** Viertler, Spasojevic & Klopfstein, sp. nov. (Fig 9)

urn:lsid:zoobank.org:act:92A2E9BC-1712-4289-8295-7DC088E1B982

#### Etymology

“Flava” meaning blonde or yellow in Latin, named after the bright yellowish body colour of the wasp.

#### Material

Holotype: male? (FUR #11112). Denmark, county: Morsø Kommune, locality: Gullerup. Leg: Rettig, Erwin. Paratype: male? (FUR #13812). Denmark, county: unknown, locality: unknown. Fur Formation, Ypresian epoch (56–54.5 mya). Leg. Rettig, Erwin.

#### Type preservation

Holotype mainly in dorsal view. Head slightly turned to the left, right antenna partly missing. Mesosoma slightly turned to the side. Traces of carinae visible on propodeum. Fore wings almost complete. Hind wings partial. Legs not clearly visible, but trace of hind leg visible on counterpart and paratype. Metasoma in dorsal view, depressed, individual tergites clearly visible. Genitalia not visible.

#### Diagnosis

See genus diagnosis.

#### Description

Body 6.1–6.9 mm (6.1 mm). Head, mesoscutum and wing venation orange to brownish. Pterostigma with dark brown spot in centre. Antenna, rest of mesosoma and entire metasoma yellowish.

*Head*. Rather short. Flagellum 5.3 mm, with around 31 segments, evenly thick throughout entire length; basal segments not clearly visible, appear at least twice as long as wide; median and apical segments slightly longer than wide. Traces of occipital carina dorsolaterally visible.

*Mesosoma*. Short and stout. Notauli seem present at least in the very front, but length and shape further back not discernible. Propodeum higher than long, with traces of carination. Legs not clearly visible, appear slender.

*Wings*. Fore wing 5–5.5 mm (5 mm). Areolet closed, quadrate-oblique, 3rs-m 1.5x longer than 2rs-m, 4M very short. 2m-cu evenly curved outwards, with two bullae that cover together around 35% of total 2m-cu length. M&RS vein strongly curved. 4Cu about 1.4x 5Cu. 4Rs straight. Ramulus absent. 1cu-a interstitial to 1M + 1Rs and intersection thickened; 1cu-a emerges in a right angle. Pterostigma 2.7x as long as wide, 1.1x length of vein 1R1. Cell 2R1 3.2x as long as wide. 2Cu 0.85x 1M + 1Rs, 1.45x r-rs. 1m-cu&2-Rs+M vein angled. 3Cu clearly longer than 2cu-a. Hind wing, 1RS 0.8x rs-m. 1Cu slightly longer than cu-a.

*Metasoma*. Dorsoventrally compressed. T1 gradually widening and 1.14x as long as wide posteriorly; spiracle in the anterior half; traces of latero-median carinae reach more than half length of T1, parallel to slightly diverging. T2 0.58 as long as posteriorly wide. T2–T4 constantly widening, with and after T5 tergites narrower again, T7 appears very short.

**Orthocentrinae** Förster, 1869

*Entypoma*? Förster, 1869

**Fig 10:**
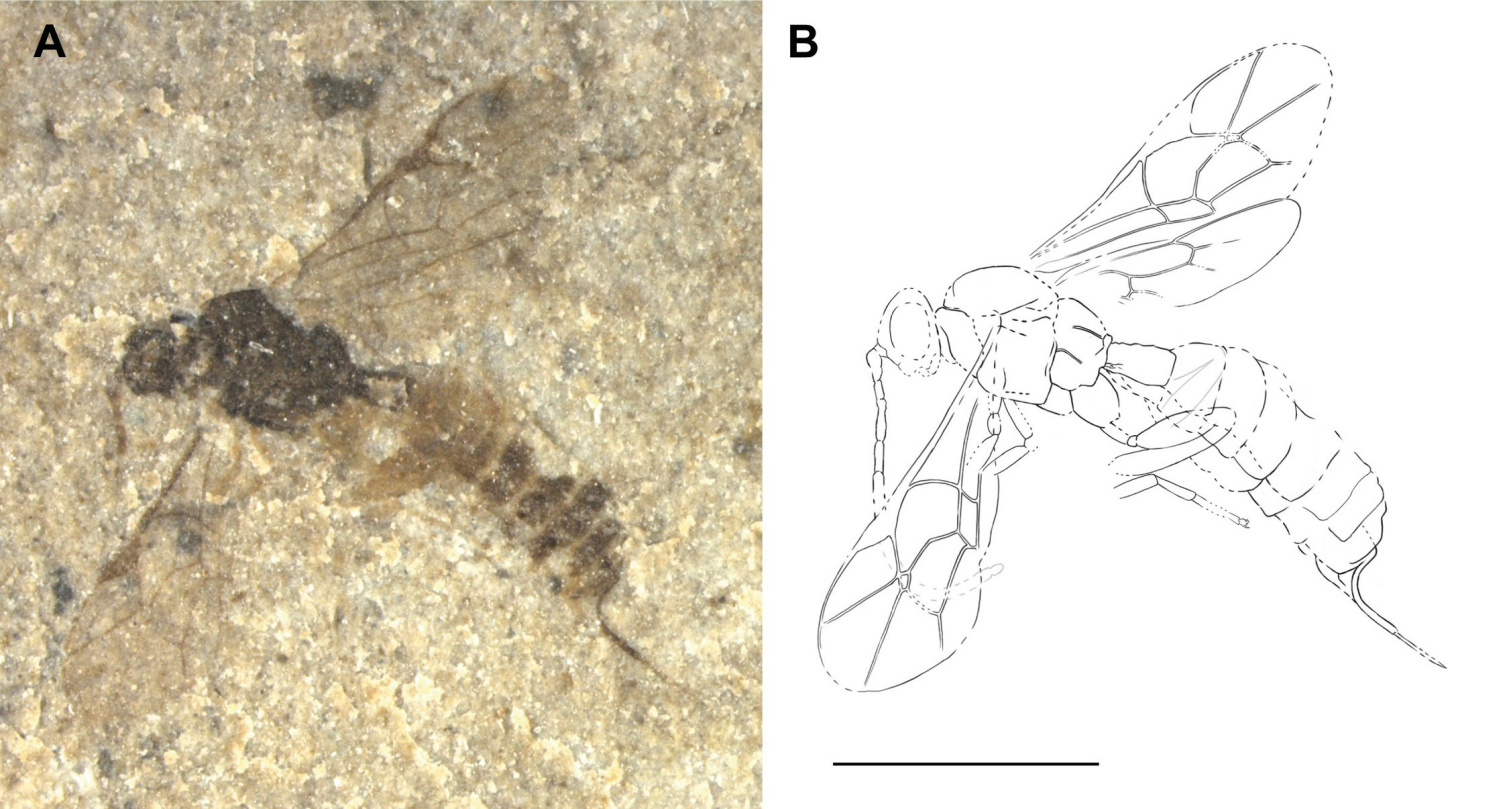
Holotype of *Entypoma*? *duergari* sp. nov. (FUR #11264). (A) Photo of the fossil part. (B) Interpretative drawing, fossil part and counterpart were used as templates. Scale bar: (B) 2mm.

***Entypoma*? *duergari*** Viertler, Spasojevic & Klopfstein, sp. nov. (Fig 10)

urn:lsid:zoobank.org:act:5D05BA68-9012-4B64-B9CD-31A7BBBFCEC6

#### Etymology

Because of the small size of this species, its name alludes to Duergar, subterranean dwarves from the tabletop game Dungeons and Dragons.

#### Material

Holotype: female (FUR #11264). Denmark, county: Morsø Kommune, locality: Klinten ved Klitgård. Leg: Rettig, Erwin. Paratype: female (FUR #11208). Denmark, county: Morsø Kommune, locality: Klinten ved Klitgård. Fur Formation, Ypresian epoch (56–54.5 mya). Leg. Rettig, Erwin.

#### Type preservation

Holotype preserved in dorso-lateral view. Antenna partially visible on counterpart. Traces of carination visible on propodeum. Fore wings almost complete. Hind wing partially discernible. Fore and mid legs not well preserved but hind leg almost complete with tarsal claw. Metasoma complete with preserved ovipositor and sheaths. Paratype preserved in dorsal view, almost complete, with antennae, hind wing and ovipositor partially discernible.

#### Systematic placement

The fossil’s characteristics, especially the small size, outward bowed 2m-cu and box-shaped T1 lead to Orthocentrinae, Pimplinae, or Tryphoninae as possible subfamilies. Orthocentrinae include some of the smallest species in Ichneumonidae, but also taxa in Pimplinae and Tryphoninae can have a rather small size and share parts of the rather large morphological variation found in Orthocentrinae. Many distinct characteristics that most Orthocentrinae have, such as narrow tapered mandibles, a strongly convex clypeus and a fringe of setae on the inner side of the hind tibia, are not visible in the fossils. Nevertheless, the large laterotergite 2 would point to a placement in Orthocentrinae. Within this subfamily there are two genus-groups, the *Orthocentrus*-group and *Helictes-*group. *Entypoma*? *duergari* sp. nov. resembles the latter due to the normal size of the scape, which is elongated in the *Orthocentrus-*group. We also can exclude the *Orthocentrus-*group because they normally have a strongly convex, bulging face, which seems not to be the case in these fossils.

The classification places this fossil to 87% (Table 4) in Orthocentrinae according to fore wing venation. Furthermore, the fossil resembles taxa in the extant *Helictes*-group the most, according to the between-group PCA in Orthocentrinae. The Euclidean distances are even shorter between the fossil and the *Helictes*-group, than between the *Helictes*-group and the *Orthocentrus*-group (S5 Table). The fossils fore wing venation does resemble the mean shape of Orthocentrinae in having a relatively short but slightly deeper 2R1 cell, an outwards bowed 2m-cu, and vein 4Cu longer than 5Cu, and the pterostigma slightly enlarged (Fig 6).

Within Orthocentrinae, there are several genera that share body features with the fossil. Since the fossils possess a closed areolet and a rather long ovipositor, we can exclude some genera. *Dialipsis* Förster, 1869, *Plectiscidea* Viereck, 1914, *Eusterinx* Förster, 1869 and *Blaptiscus* Thomson, 1892 have their T1 fused with S2 and rather narrow and elongate, which does not match with the box-like T1 of these fossils. *Aperileptus* Förster, 1869 and *Entypoma* Förster, 1869 resemble this fossil by having two bullae on 2m-cu and the apex of S1 before the middle of T1. Furthermore, in the hind wing, 1Cu is longer than cu-a in our fossils, which speaks for a placement in *Entypoma*, since in *Aperileptus*, the vein 1Cu & cu-a is not intercepted. The probable crease that separates the epipleura of T2 and T3, and the rather depressed metasoma increase a possible affiliation to *Entypoma*. Nevertheless, to confirm the placement in *Entypoma*, we would need more details of the face and the mesosoma, like a small, rather flat clypeus with narrowing and twisted mandibles, and long notauli that reach to the centre of the mesoscutum. We thus express the uncertainty in the generic classification by adding a question mark.

#### Diagnosis

There are seven other described fossils from the *Helictes-*group in Orthocentrinae, the oldest being *Lithotorus cressoni* Scudder, 1890 from the Green River Formation [36]. This species has a clearly pentagonal areolet and shorter 2R1 cell in the fore wing. The three species from Baltic amber [47] all have a very slender and elongate T1. Finally, there are three species classified in *Eusterinx* from the Late Eocene locality on Isle of Wight [48]. While two of them have a very elongate T1 and elongate fore wings that clearly distinguish them from our fossil species, *Eusterinx vectensis* (Cockerell, 1912) possesses a large closed areolet and might have been placed in the wrong genus, since it lacks the metasoma and thus cannot be interpreted. With the visible characteristics it could as well belong to *Entypoma*, but it differs from the present fossil by the straight 2m-cu in the fore wing. *Entypoma*? *duergari* sp. nov. differs from extant *Entypoma* taxa by its small size and weak or absent notauli.

#### Description

Body 4.4–4.8 mm (4.4 mm). Head, mesosoma and T1 dark brown. Antenna, legs, T2 and T3 seem yellow/orange. T 4–8 and ovipositor sheaths brown.

*Head*. Short and rather small. Flagellum 2.5–3 mm (2.5 mm), number of segments unclear but at least 16; evenly thick throughout whole length. Temple appears narrow.

*Mesosoma*. Rather short and stout. Notauli either absent or weak; if present then not extending beyond middle of mesoscutum. Mesopleural furrow more or less straight. Metapleuron about as long as wide. Pleural carina present; unclear if reduced posteriorly or complete. Propodeum evenly rounded in profile, about as long as high; with longitudinal carinae present, other carinae unclear. Legs seem slender. Hind leg with coxa of normal dimension, slightly longer than wide; femur 3.2x as long as wide; tibia dimensions normal; tarsal claws appear simple.

*Wings*. Fore wing 2.9–3.7 mm (2.9 mm). Areolet closed, quadrate-oblique, 4M short, 2+3M and 3rs-m similar in length. 2m-cu slightly and evenly curved outwards, with two bullae, which are covering about 36% of total 2m-cu length. 4Cu 1.8x 5Cu. 4Rs straight. Ramulus absent. 1cu-a interstitial to 1M + 1Rs, with 1cu-a bowed distally. Pterostigma about 2.5x as long as wide; 0 .8x 1R1. Cell 2R1 2.3x as long as wide. 2Cu 0.83x 1M + Rs, 1.1x r-rs. 1M + Rs 1.3x r-rs. 1m-cu&2-Rs+M angled. 3Cu clearly longer than 2cu-a. Hind wing with M + Cu slightly curved distally; 1Cu longer than cu-a, last abscissa of RS and CU seem mostly transparent.

*Metasoma*. Depressed or cylindrical. Sclerotized part of S1 seems not to reach spiracle, apparently free from T1. Laterotergite 1 indistinct. Laterotergites 2, 3 and 4 rather broad, about 0.4–0.5x as wide as long; appear to be separated from dorsal part of tergite by a crease. T1 2x as long as posteriorly wide, box-like; with spiracle in anterior half; T2–T7 similar in length, very short and wide. Ovipositor around 0.38x length of metasoma; ovipositor and sheaths seem parallel-sided; shape of tip unclear.

**Ctenopelmatinae?** Förster, 1869

Perilissini? Thomson, 1883

Lathrolestes? Förster, 1869

**Fig 11:**
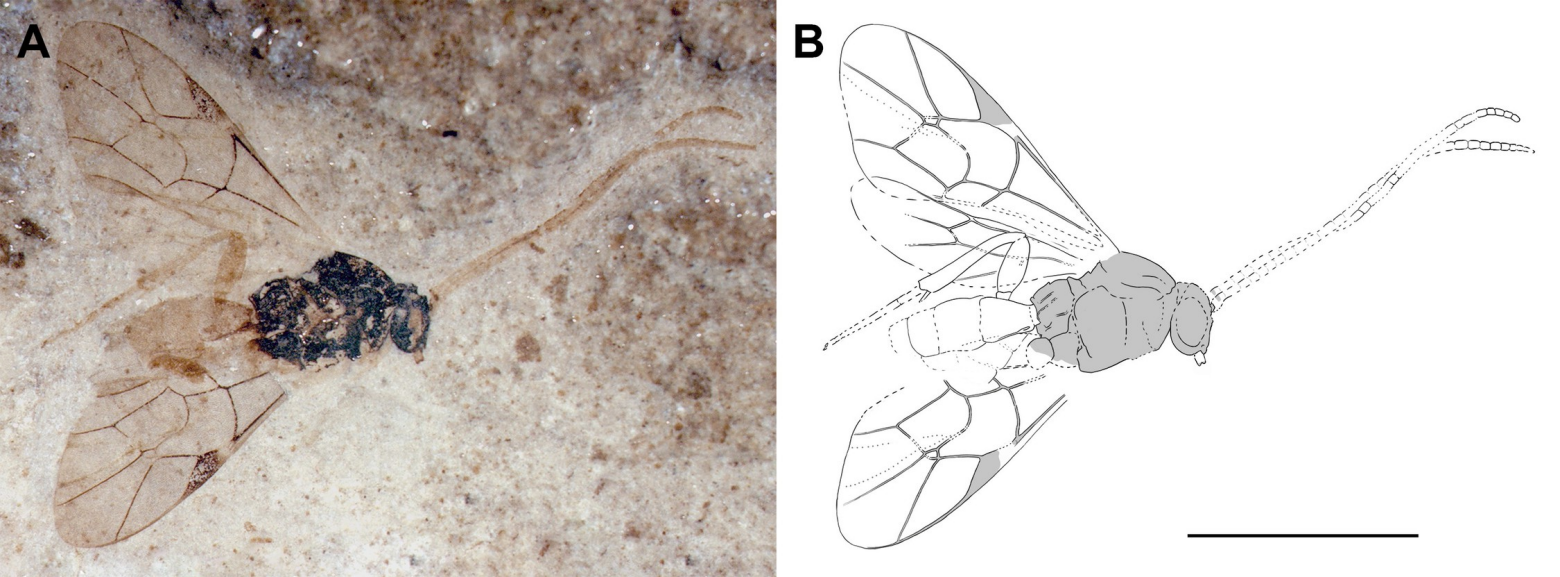
Holotype of Lathrolestes? zlatorog sp. nov. (FUR #10651). (A) Photo of the fossil part. (B) Interpretative drawing, fossil part and counterpart were used as templates. Scale bar: (B) 5mm.

***Lathrolestes*? *zlatorog*** Viertler, Spasojevic & Klopfstein, sp. nov. (Fig 11)

urn:lsid:zoobank.org:act:508DC4A4-0549-483C-8C43-FF92DDA53DBE

#### Etymology

Named after Zlatorog, a chamois buck with golden horns from a Slovene folklore, due to the yellowish bright and long antennae.

#### Material

Holotype: male? (FUR #10651). Denmark, county: Morsø Kommune, locality: Ejerslev. Fur Formation, Ypresian epoch (56–54.5 mya). Leg: Verkleij, Jan.

#### Type preservation

Holotype in lateral view. Antenna well preserved, but single segments indeterminable. Head in profile with visible mandible. Mesosoma well preserved, propodeum shows traces of carination. Fore wings almost complete. Partial hind wings preserved, base indiscernible. One hind leg well preserved with apical spurs and tarsal claw. Metasoma only partially preserved, from T1 to T3 or T4. Genitalia not visible.

#### Systematic placement

The combination of a rather stout appearance, depressed metasoma with a sessile, stout first tergite and an oblique quadrate areolet with a short stalk leads to some Banchinae, and most Tryphoninae and Ctenopelmatinae. However, the longitudinal carina on the propodeum would exclude Banchinae, and also the bright antennae are rather rare in this subfamily.

When trying to place the fossil in a candidate subfamily, the result of the cross-validated CVA assigns a 32% probability for this fossil to belong in Ctenopelmatinae and a 68% probability to belong to Tryphoninae (Table 4). These two subfamilies are difficult to distinguish in general, but also their fore wing venation seem very similar according to their Procrustes distances (Fig 4, S3 Table). The similarity of Tryphoninae and Ctenopelmatinae are also visible in the meanshapes, with one of the few differences being the slightly broader 2M and 3M cells in Tryphoninae (Fig 6). In the between-group PCA the two closest groups to the fossil fore wing are two tribes in Ctenopelmatinae: Perilissini and Pionini. But the tribes Tryphonini, Oedemopsini and Exenterini, belonging to Tryphoninae, are also similar to the fossil fore wing. In this fossil the classification analyses did not provide more insight in a distinct subfamily classification, but we compared the fossils body characteristics, including wing characteristics that are not covered with our geometric morphometric approach, with the closest resembling tribes of the bgPCA results. And since the straight shape of the 2m-cu with only one larger bulla (Fig 7) and the very long and brightly coloured antennae does rather point to Ctenopelmatinae, we focused our comparison within those subfamilies tribes, but indicate the uncertainty of this placements with questions marks.

Many characteristics to identify the fossil further are not visible, but the features we do see match taxa of the tribe Perilissini, which usually possess a deep glymma, also visible in this fossil. Also, the wing venation of the fossil matches Perilissini taxa, especially the shapes of the 2m-cu and 1m-cu&2-Rs+M veins and the emerging of the r-rs vein of the pterostigma.

Within the Perilissini, the fossil shares some characteristics with the genera *Perilissus* Förster, 1955 and *Lathrolestes* Förster, 1969, for example a flat to slightly convex clypeus in profile, the lower tooth of the mandible a little longer than the upper, vein M+Cu in the fore wing only weakly arched, and 1Cu in the hind wing about the same length as cu-a. The deep but rather short glymma and the T1 that is around 1.65x as long as wide and only weakly arched, rather point to *Lathrolestes* than *Perilissus*. To place the fossil with certainty in *Lathrolestes*, more characteristic details and a complete preserved metasoma would be necessary.

#### Diagnosis

There are only four described fossil species in Ctenopelmatinae and none of them are in the genus *Lathrolestes* or appear similar to this fossil. *Lathrolestes*? *zlatorog* sp. nov. differs from extant *Lathrolestes* by its rather flat first tergite in dorsal view and its simple tarsal claws on the hind leg.

#### Description

Body 6.8 mm. Head and mesosoma dark brown. Mandibles, antenna, legs and metasoma bright orange. Wing venation dark brown.

*Head*. Mandible bidentate; lower tooth a little wider and longer than upper tooth; both tips appear evenly rounded. Malar space about 0.8x mandibular width. Face including clypeus flat in profile. Flagellum 8 mm, with at least 30 segments, evenly thick throughout whole length, central flagellar segments about 1.2x as long as wide. Temple seems short.

*Mesosoma*. Pronotum about 0.6x as long as high. Notauli either absent or very weak. Epicnemial carina laterally and ventrally present. Propodeum rounded in profile; about as long as high; lateromedian and lateral longitudinal carinae present; anterior and posterior transverse carinae indiscernible; hind margin appears simple. Hind leg with coxa of normal proportion; femur 3.2x as long as wide; tibia normal, evenly tapering, with one tibial spur visible but the second one potentially hidden; tarsal claw appears simple.

*Wings*. Fore wing 7.4 mm. Areolet closed, quadrate-oblique, with 4M about half the length of 2Rs. Upper half of 2m-cu straight, then bowed outwards, with one large bulla covering 45% of whole 2m-cu vein. 4Cu 1.5x of 5Cu. 4Rs almost straight. Ramulus absent. 1cu-a postfurcal to 1M + 1Rs, with 1Cu about as vein width of 1cu-a; 1cu-a emerging distally. Pterostigma 2.9x as long as wide. Cell 2R1 2.6x as long as wide. 5M tubular throughout whole length. 2Cu 0.7x 1M + 1Rs and 0.9x r-rs. 1m-cu&2-Rs+M arched. 3Cu almost double the length of 2cu-a. Hind wing only partially visible, with 1Rs 1.5x rs-m.

*Metasoma*. Depressed until T4, rest unclear. T1 about 1.25x longer than wide; evenly tapering to front; spiracle in anterior half; with trace of dorso-lateral carina. T2–T4 about 1.2x wider than long.

**Metopiinae** Förster, 1869

*Triclistus* Förster,1869

**Fig 12:**
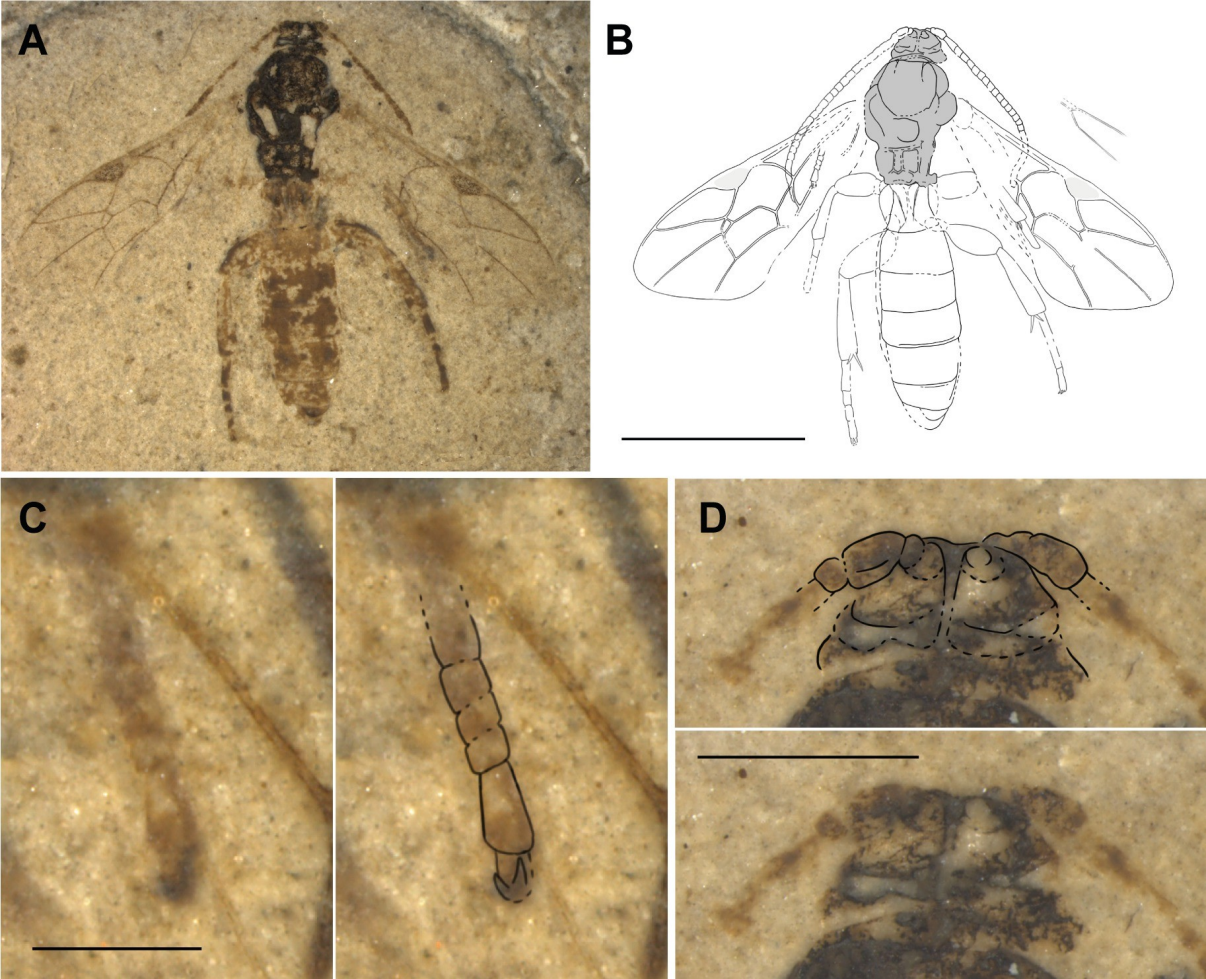
Holotype of *Triclistus bibori* sp. nov. (FUR #13809). (A) Photo of the fossil part. (B) Interpretative drawing, fossil part and counterpart were used as templates. (C) Photo and drawn outlines of the fore tarsus, with the tarsomeres 2–4 very short and stout. (D) Head with visible groove between antennae. Scale bars: (B) 4mm, (C) 1mm, (D) 0.5mm.

***Triclistus bibori*** Viertler, Spasojevic & Klopfstein, sp. nov. (Fig 12)

urn:lsid:zoobank.org:act:544F7D34-E61A-4862-9247-40612C518DEE

#### Etymology

Derived from the German name of the wasp Pokémon Bibor.

#### Material

Holotype: male? (FUR #13809; see also remarks below). Denmark, county: unknown, locality: unknown. Leg: Rettig, Erwin. Paratype: male? (FUR #11215). Denmark, county: Morsø Kommune, locality: Klinten ved Klitgård. Leg: Rettig, Erwin. Paratype: male? (FUR #11218). Denmark, county: Morsø Kommune, locality: Klinten ved Klitgård. Leg: Rettig, Erwin. Paratype: male? (FUR #13077). Denmark, county: Morsø Kommune, locality: Svalklit. Fur Formation, Ypresian epoch (56–54.5 mya). Leg: Rettig, Erwin.

#### Type preservation

Holotype in ventral and dorsal view, part more dorsal and counterpart more ventral. Apical part of both antennae mostly missing in part, well preserved in counterpart. Mesosoma rather well preserved, propodeum with carination. Fore wings almost complete. Hind wings, except vein 1Rs and 2Rs, indiscernible. All legs rather well preserved on counterpart; especially hind coxae prominently visible. Metasoma distinctly preserved. Genitalia not well visible, hard to interpret.

#### Systematic placement

The character combination of these fossils strongly resembles Metopiinae. As the extant species in this subfamily, the fossils possess a compact body shape, a high lamella between the antennal sockets, short notauli, short and stout legs, shortened tarsal segments in the fore legs, and the first tergite short and stout with the spiracle on the anterior half, and with strong median longitudinal carinae.

The parallel-sided flagellum, the deep groove on the head between the antenna, and the mid and hind tibia with two apical spurs, are typical for species in the genera *Colpotrochia* Holmgren, 1856 and *Triclistus*. The distinct carination on the propodeum and the broad and stout T1 in the fossils exclude *Colpotrochia* as a possible genus. The strongly postfurcal 1cu-a to 1M + 1Rs, the broad T1 with two median longitudinal carinae and the stalked areolet are highlighting the resemblance to *Triclistus*.

Because the body characteristics of this fossil so strongly point to this subfamily, we tested it together with two other ophioniform subfamilies, Campopleginae and Ctenopelmatinae in the geometric morphometrics analysis. Surprisingly, according to the fore wing venation in the classification analysis, the fossil holotype does not cluster with extant Metopiinae, where we removed three landmarks due to missing information (Table 1). Instead, the subfamily assignment places the holotype closest to Ctenopelmatinae, followed by Campopleginae. Indeed, the large distance between the wing venation of *T*. *bibori* sp. nov. and extant Metopiinae is somewhat at odds with the very similar morphology of the rest of the body. However, none of the wing characteristics speak clearly against Metopiinae, and it might be possible that the removed LMs (LM 18 and LM 19) are very informative for this subfamily, as we see changes in the mean shapes (Fig 6). We thus also tested two of the paratypes (#11215 and #13077), which possess almost complete fore wings and for which only one landmark had to be removed (Table 1). Both paratypes classify in Metopiinae. Looking at the mean shapes of the subfamilies and the fossil holotype, the slightly enlarged pterostigma would not point to Metopiinae, but the strongly postfurcal 1cu-a and the clearly longer 3Cu in comparison with 2cu-a matches well (Fig 6). In the between-group PCA, when considering the tribes of Ctenopelmatinae, Campopleginae, *Metopius* and other Metopiinae, the paratypes resemble Metopiinae (without *Metopius*), but also Perilissini in Ctenopelmatinae. In the same analysis, but within Metopiinae only, the holotype and both paratypes are not only similar to each other, but also to *Triclistus*.

Considering the most complete fore wings of the paratypes, together with all other body characteristics of the fossils, the systematic placement clearly leads to *Triclistus* in Metopiinae. These fossils represent the oldest and only second fossil representative of *Triclistus*, the other one being *Triclistus ventrator* Khalaim, 2008 [49] from the Oligocene of Biamo, Russia.

#### Diagnosis

*Triclistus bibori* sp. nov. differs from *T*. *ventrator* by body length, with *T*. *bibori* sp. nov. being almost twice the size. Furthermore, the areolet in this fossil is not as strongly petiolate and unlike *T*. *ventrator*, *T*. *bibori* sp. nov. possesses two spurs on the hind tibia, where one is relatively long and the other about half the length. But maybe the second tibial spur on *T*. *ventrator* is missing due to poor preservation. Another difference to distinguish the two *Triclistus* fossils, but which may also be an artefact of preservation, is the colouration of the metasoma, which seems dark brown to almost black in *T*. *ventrator* and bright orange/brown in *T*. *bibori* sp. nov. From extant species of the genus, *T*. *bibori* sp. nov. can be distinguished by the generally stouter flagellomeres, in combination with a large areolet and a rather short propodeum, which is usually more elongate in extant taxa.

#### Description

Body 7.5–8.8 mm (8.5 mm). Head and mesosoma dark brown. Antenna and wing veins orange to brownish. Legs and metasoma orange. Paratypes with somewhat lighter colouration, maybe due to surrounding rock.

*Head*. Rather short. Scape a little longer than wide. Pedicel smaller than scape. Flagellum 4.0 mm, with at least 25 segments, evenly thick throughout whole length; segments rather stout, most 1.0–1.2x as long as wide. Lamella between antennal sockets reaching to around height of median ocelli with parallel deep grooves.

*Mesosoma*. Rather stout. Notauli distinct but rather short. Propodeum short, with distinct carination, pleural carina partially visible, as well as posterior transverse carina, lateromedian longitudinal carina and lateral longitudinal carina. Legs short and stout. Tarsal segments 2–4 shortened in front leg. Two tibial spurs on mid tibia, similar in length. Hind femur medially swollen. Hind tibia evenly tapered to apex; with two spurs, outer spur half as long as inner spur. Tarsal claws appear simple.

*Wings*. Fore wing 5.7–6.3 mm (6.2 mm). Areolet closed, quadrate-oblique, 3rs-m with one bulla and almost meeting 2m-cu directly, 4M nearly obliterate. Vein 2m-cu straight with one bulla, which is covering 35% of total 2m-cu length. 4Cu 1.2x 5Cu. 4Rs almost straight. Ramulus absent. 1cu-a postfurcal to 1M + 1Rs. Pterostigma 2.4x as long as wide, 0.8x length of vein 1R1. Cell 2R1 2.8x as long as wide. 2Cu 0.82x 1M + 1Rs, same length as r-rs. 1M + 1Rs 1.2x r-rs. 1m-cu&2-Rs+M angled. 3Cu clearly longer than 2cu-a. Hind wing indiscernible.

*Metasoma*. Dorsoventrally compressed. T1–T6 wide, relatively short. T1 very stout, appears parallel sided, about 0.9x as long as wide, with strong median longitudinal carina, reaching more than half length of tergite. T2–T6 about 0.5x as long as wide. T7 appears partially retracted. Genitalia not visible.

#### Remarks

Females in the genus *Triclistus* Förster, 1869 possess a characteristic hook on the last tarsomere of the hind leg. Additionally, S6 in females is elongated, with an apical rounded notch and T7 is completely retracted. Our fossils appear to have no hook on the tarsomere, and a roundish, mostly partially retracted metasomal apex, which leads to the assumption of our fossil specimens of *T*.? *bibori* sp. nov. being males.

## Discussion

### Robust data quality revealed

Our data quality evaluations showed negligible differences when different people place landmarks, which confirms previous investigations of this aspect in other studies [54, 55]. We could also demonstrate that the differences between forewing drawings from the literature and photographs of flattened wings were minor, at least if the former were obtained in a standardised fashion similar to the one used by Townes [36–38, 46]. This also suggests that photographs of different species could easily be added to our dataset in the future. This is at least the case if the dataset is used for classification purposes above the genus level, as some of our replicates using different congeneric species resulted in a partial overlap of intra- versus inter-generic distances (Fig 2A). This was especially the case in some large genera and might partly be due to non-monophyly of the involved genera; indeed, for instance the species included in *Bathyplectes* used to be classified in two different genera in the past [17]. In any case, while the limits of our approach for classifying taxa at or below genus level requires further scrutiny, it appears very robust for higher-level classification [10, 11].

Fossilisation artefacts appear to only have a minor effect on the performance of the method, at least if the wings appear reasonably flat, which is the case in a large portion of compression fossils. In our analysis, the fore wings of part and counterpart, as well as the left and right side of a compression fossil, which often showed a slightly different quality of preservation, were very similar. This indicates that it does not really matter which wing is used in the geometric morphometric analysis, except if it is obviously distorted or damaged. This insight is especially crucial for palaeontology, as preservation of compression fossils can vary widely [32].

### Wing size and shape correlation

Fore wing shape is strongly correlated with centroid size in our dataset (Fig 3), with the aspect ratio (length to width) increasing with size. This allometric relationship means that smaller species tend to have relatively wider, and larger species relatively more narrow wings, which might ensure a minimum surface area of the wing conferring sufficient uplift even in small species. Our finding is in agreement with previous studies that found that fore wing length is a good proxy for specimen size [56, 57], while fore wing width did not seem to be strongly correlated to size, at least in Apoidea [58]. However, the relationship of size and aspect ratio varies among insect groups, which might be due to differences in other aerodynamic properties of the wings [59].

The wing cells of Darwin wasps tend to change in parallel with the entire wing, becoming more narrow in larger species. This effect is strongest for the central and apical cells (Fig 3). This leads to a rather even distribution of wing veins over the entire length and width of the wing in larger specimens, which might stabilise the wing in larger species. A similar pattern, including the increasing pterostigma width in smaller specimens, has been observed in other Hymenoptera as well [59, 60], although considerable variation has been observed in other insect orders [59].

We did not observe any reduction of wing veins in small-bodied species in our dataset, as it is observed in many other groups of Hymenoptera [61]. Indeed, the wing venation of Darwin wasps is surprisingly constant, with most veins present even in the smallest representatives of the family [21]. However, there are two exceptions, Hybrizontinae and Neorhacodinae, both rather small-bodied and species-poor subfamilies that were not included in our dataset. In both cases, the reduction mostly concerns the apical part of the wing, and the remaining veins furthermore shift towards the base [21].

Interestingly, the allometry effect varies among some of the subfamilies, despite some of them sharing the same size range, e.g. Cremastinae and Tryphoninae. Ophioninae stand out in this respect by exhibiting a rather large fore wing size range, but only minor allometric effects. Most Ophioninae species are nocturnal, and wing features of nocturnal Hymenoptera also seem exceptional in other lineages [15, 62], although the reason for this observation remains unknown. Irrespective of their body size, Ophioninae wings all appear rather similar to those found in previous analyses of larger hymenopterans, which possess a slender pterostigma and more elongate distal cells, whose functions appear related to flying performance [62, 63]. Overall, the varying correlations of fore wing size and venation shape observed among many subfamilies might be caused by more than one factor. Evolutionary history, geographical components, hosts, selective pressures in response to mimicry, and body shape variation all are factors that might influence shape and size differences [56, 64]. To disentangle the reasons for the different allometry patterns among subfamilies, one needs to be able to rule out environmental or genetic variation that affect size, or to know the extent of their influence in order to correct for it [65].

### Variation within and between extant subfamilies

According to the Procrustes variance within the subfamilies, the most extensive shape variation is found in Metopiinae and Cremastinae, whereas the least variance was found in Tersilochinae and Campopleginae (Fig 4, S4 Table). Why fore wings of Cremastinae are the most variable among the included subfamilies is unclear, given that the subfamily is rather well-defined morphologically, with the exception of the *Belesica* group of genera, and has consistently been recovered as monophyletic in phylogenetic analyses [20, 66]. Their wings not only vary in cell dimensions, but also in areolet shape and pterostigma dimensions, although their short and straight 2m-cu is very consistent. Ecology as a possible explanation for this large fore wing variation seems unlikely, since they all do share similar habitats and hosts. The hosts might be concealed differently, so the available space for their pupation might differ, but this is highly speculative. In Metopiinae, the variation is introduced mostly by the genus *Metopius*, which is represented by seven species in our dataset and exhibits a much narrower fore wing and a clearly different areolet shape from the other Metopiinae genera (Fig 7). *Metopius* species are also distinct in other body features, like having a shield-like face, only one mesotibial spur and being generally larger than most other Metopiinae genera [67]. They also are exceptional among other members of the subfamily in attacking free-ranging Lepidoptera, instead of hosts that are hidden in silk webbing or leaf-rolls [21], so the difference in wing venation might be due to this environmental factor or the size difference.

Wing characteristics like the shape and length of the cell 2R1, the emerging position of 1cu-a, the shape of the areolet and many more have previously been used in identification keys [21, 68]. It is therefore surprising that this is the first geometric morphometric approach to distinguish ichneumonid subfamilies. While other geometric morphometric studies successfully discriminated taxa at lower taxonomic ranks [69–71], we here demonstrate that fore wing venation is sufficient to distinguish many extant ichneumonid subfamilies (Figs 4 and 5), with shape differences usually larger between subfamilies than within them (Fig 4, S4 Table). Many subfamilies can thus be reclassified successfully in the CVA, although some remain difficult to distinguish, especially Ctenopelmatinae and Tryphoninae (Table 3). The classification accuracy could probably be increased by adding more species to each subfamily, considering that there are more than 25’000 described ichneumonid species [17]. Subfamilies like Pimplinae and Ctenopelmatinae are probably not even monophyletic [20, 21, 72], although at least in Pimplinae, this did not have a noteworthy impact on the success of their classification; this might be linked to the clade’s wing venation being rather plesiomorphic in many aspects [30]. In Ctenopelmatinae, the classification success was often low, especially when compared with Tryphoninae (Table 4), and it might improve once the subfamily is split into monophyletic entities. The similarity between Ctenopelmatinae and Tryphoninae was already observed in many body characteristics before [21, 68], even though they are not very closely related within Ophioniformes; they are thus an excellent example that morphoclustering can differ from phylogeny because of plesiomorphic characters and divergent rates of evolution [73]. While Tryphoninae is probably the sister group of the rest of Ophioniformes [20], Ctenopelmatinae might just have maintained the plesiomorphic fore wing venation in parallel. Plesiomorphic trait retention, natural and sexual selection, genetic drift, and abiotic factors might change wing venation faster than visible in molecular data, as seen in fossil halictid bees [11]. Convergence could be another explanation, and it underlines the importance of not confusing morphoclustering and phylogenetics. Ctenopelmatinae and Tryphoninae share the same hosts, sawfly larvae [37, 68], which makes an ecological influence on morphology probable. But this would need further investigations, and the complexity of influencing factors needs to be kept in mind. Despite such effects, the high classification success observed in our study at the tribal and subfamily level suggests that there is a lot of phylogenetic information in wing venation waiting to be harvested even for deep phylogenetic studies in Darwin wasps.

### Fossil classification aided by geometric morphometrics

Our study shows that in ichneumonids, geometric morphometrics of fore wings results in robust leads to possible affiliations of fossil species to extant subfamilies. Quantifying the shape similarity of fossil specimens with extant taxonomic groups by use of geometric morphometrics is a rather new application, but it has already proven successful in various taxa. For example, in a fossil agamid lizard (Amphibolurinae), the jaw was used to compare it with extant genera [74]. In bees and wasps, the fore wings have repeatedly been used to assign fossils to extant groups, for example in fossil bumble bees (Apidae) to tribes or subgenera [13, 75], halictid bees (Halictidae) to tribes [11], and in ensign wasps (Evaniidae) to genera [16]. Using geometric morphometrics to inform fossil classification is certainly a very promising approach and will gain further traction in the near future.

However, we also encountered some potential pitfalls of the method. For instance, in *Triclistus bibori* sp. nov., the classification analysis gave ambiguous results, which might be due to the fact that three landmarks were missing in the holotype. This highlights the necessity of the fore wings to be as complete as possible, since too much information could get lost with only a few missing landmarks. Alternatively, the problem might also be explained by the relatively small number of extant Metopiinae taxa in our analysis (N = 28); this would be difficult to rectify, as the subfamily does not include many more genera. Finally, our ambiguous classification result might also be an indication of an especially fast evolution of wing venation in Metopiinae, which is somewhat supported by the large difference between *Metopius* and the remaining genera in the subfamily. Indeed such a speed-up of evolution could be expected to result in a pronounced difference between fossil and extant taxa, but this remains to be shown and would need further investigation.

Another potential pitfall relates to the fact that we attempted to classify the new fossil taxa into existing groups, in our case extant subfamilies, or even further to extant tribes if possible. Such an approach might however be unjustified for really old fossils, especially if there was a high turnover of higher taxa through time. In such cases, the fossils might instead belong to a stem lineage and not a crown group, or even to an extinct clade that has not yet been discovered and named. Indeed, if the Euclidean distances in the between-group PCA were very large between the fossil specimen and the given taxonomic groups, it might be due to the fact that we did not include the closest relatives yet, which might be already extinct. For example, the distances between *Ebriosa flava* gen. et sp. nov. and many tribes of Ctenopelmatinae or Tryphoninae are larger than the distance between the tribes of those two subfamilies. The exceptional fore wing venation of *Ebriosa flava* sp. nov., with the strongly curved M&Rs vein, raises the possibility of it belonging to an extinct tribe, a stem-lineage of either Tryphoninae or Ctenopelmatinae, or even to a now extinct subfamily that branched off even earlier. Classifying the many remaining undescribed ichneumonid fossils from the Fur Formation and integrating fossils from other formations of similar age, such as the Menat Formation in France [76, 77] might help shed light on alternative groupings the fossil might belong to. Also, integrating the relevant wing vein characteristics into a proper phylogenetic analysis would help distinguish between a stem position of a fossil in the tree of extant taxa and its belonging to a now extinct lineage, a problem that cannot be easily solved by similarity-based methods.

Phylogeny-based methods in general would provide the means of making the most of the information inherent in insect wing venation, while avoiding some of the pitfalls of similarity-based methods. However, it remains somewhat unclear how to best extract relevant characters for phylogenetic analyses from geometric morphometric datasets [78, 79]. Methods to estimate the phylogenetic signal in GM data, on the other hand, are already rather well established [80], and they could be used to separate the prevalent noise inherent in morphometric data from the signal due to deep phylogenetic relationships. Such approaches could also be used to aid placing fossil specimens, at least if an adequate phylogeny of the group is at hand.

## Conclusions

With this work, we provide an example of how to use geometric morphometric approaches on fossil wings in order to identify possible affiliations to extant groups. We demonstrate that the method can be used successfully at higher taxonomic levels, as well as in mega-diverse taxa such as Darwin wasps, at least if combined with a careful *a priori* choice of candidate subfamilies. It would be interesting to test whether such a preselection of potential groups is strictly necessary, or if the information inherent in wing venation might in some cases even be sufficient for higher-level placement, both in Darwin wasps and in other insect groups.

With more than 25,000 described extant species and only about 300 described fossil species, we can be certain that there is still much to be discovered in the ichneumonid fossil record. It is crucial to describe more fossil species to disentangle and estimate the true richness of Darwin wasps in different geological epochs. As we demonstrated here, geometric morphometrics can be used to support a robust classification of these fossils, and our assembled dataset provides a basis for future comparative analyses in ichneumonid fore wing venation not only in fossils, but also in extant species. It would definitely be worthwhile to add additional fore wing data for as many groups as possible, including other subfamilies and additional fossil taxa from different formation sites, to disentangle the considerable fore wing variation found in these wasps. Our dataset is not only of help for fossils from the Fur Formation but might also be applied to ichneumonid fossils from other formations, including amber inclusions and of course fossils from the Cretaceous. As soon as a robust phylogeny of the family is at hand, it would be interesting to use it to reconstruct ancestral shapes of fore wings, as it was done before in Braconidae, the sister group of Ichneumonidae [81]. This may lead to explanations of the evolutionary changes in wing venation and their potential causes, such as size, ecology, and/or phylogeny.

In a next step, extracted wing shape information could also be incorporated into phylogenetic analyses, although it remains somewhat unclear how to best extract the most relevant aspects of Darwin wasp wing venation for deep phylogenetics, while avoiding adding too much noise to the analysis. Our analyses indicate that ichneumonid fore wings harbour a lot of information even on phylogenetic relationships that date back over a hundred million years ago [82], and a lot of this information has hitherto remained nearly untapped. Once the methodological questions have been solved, integration of wing morphology in a total-evidence phylogenetic and maybe even dating framework could thus contribute to a better understanding of the history of this parasitoid taxon through time and help elucidate the drivers behind its extraordinary species richness. It is highly likely that similarly underexplored sources of information persist in many other taxonomic groups, given that morphometrics is assumed by many authors to be too noisy to recover deep phylogenetic relationships and is thus still mostly used at comparatively low taxonomic levels [80]. While this might be true in many cases, our results indicate that it is worthwhile to challenge this notion from time to time, and to further invest in ways to separate signal from noise in morphometric datasets.

## Supporting information

S1 Fig Canonical variation analysis with nine extant subfamilies. Plot of the first two CV axes with examples of the shape at the maximum and minimum of the first two canonical axes. If subfamilies are more or less separate from others, we coloured the max/min shape of the wing accordingly. Grey wing outlines represent the general shape if several subfamilies overlap. The outlines on the wing illustrations are interpretative and oriented on the specimen that has the minimum/maximum CV score on the respective axis.
(TIF)

S2 Fig Canonical axis 5 against canonical axis 6 of seven extant subfamilies. The fore wing shown in CV5 + 3 represents a Campopleginae fore wing, the fore wing in CV6 +3 represents a Tryphoninae fore wing. The other two greyish fore wing shapes represent the most common shape, where most species cluster in the plot.
(TIF)

S1 Table Reference dataset. List of all included taxa with information of body and fore wing length in both sexes, as well as the calculated mean of those measurements, if a range was given.
(XLSX)

S2 Table Procrustes ANOVA results of all three data quality assessments. Shows the sampling for each data quality assessment and the corresponding results of the Procrustes ANOVA.
(XLSX)

S3 Table Procrustes variance and distances of extant subfamilies. The Procrustes variance within each subfamily is shown. Additionally, the mean distances between species pairs among subfamilies is listed in an ordered manner.
(XLSX)

S4 Table Regression analyses of shape and size. Results of the Procrustes ANOVA are shown, including subfamily affiliation and centroid size as variables to explain fore wing venation shape. The unpooled and pooled regression analyses are shown, as well as the corresponding classification results using their residuals, as well as the raw dataset (Procrustes coordinates).
(XLSX)

S5 Table Fossil assignment. Classification results of each new fossil specimen and results of the between-group PCA for the narrower groups of the candidate subfamilies.
(XLSX)

S1 File TPS data quality assessment 1 and 2. Data set of data quality assessment 1 and 2, including a Campopleginae subset, where landmarks were placed by two different persons (“A” represents person 1, “M” represents person 2) on illustrations and once on photographs (represented by “p”).
(TPS)

S2 File TPS data quality assessment 3. Data set of data quality assessment 3, including the landmark placement of the five new fossil specimens with part/counterpart and right/left side if available.
(TPS)

S3 File TPS reference data set. Reference data set, including 333 extant species, 9 fossil Pimplinae from the Fur Formation, the five new fossil species (holotypes) and the two paratypes of *Triclistus bibori* sp. nov.
(TPS)
