# Supplementary figures and images for: Classifying fossil Darwin wasps (Hymenoptera: Ichneumonidae) with geometric morphometrics of fore wings
Source: PLoS One. 2022 Nov 17;17(11):e0275570. doi: 10.1371/journal.pone.0275570 (PMC9671425; doi:10.1371/journal.pone.0275570)

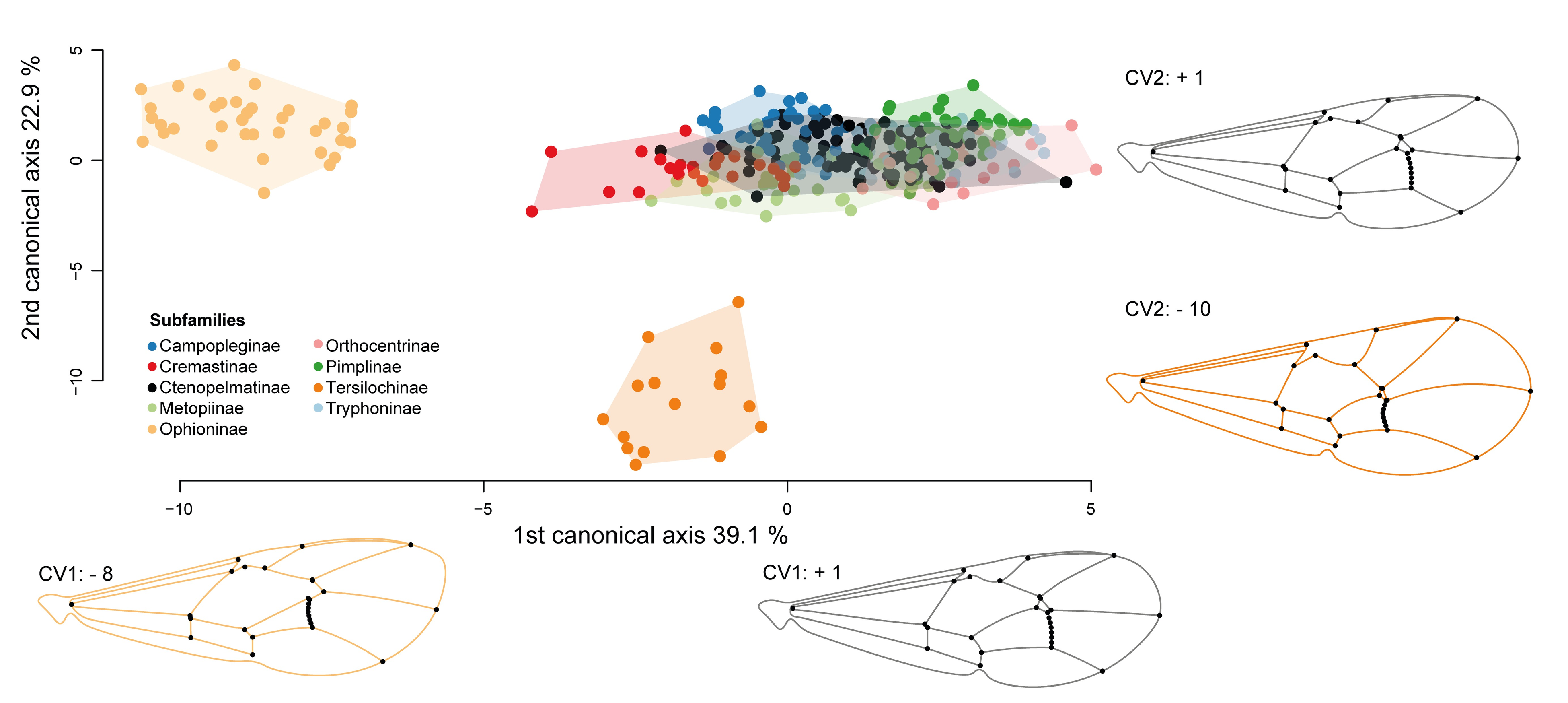

Supplement: S1 Fig — Plot of the first two CV axes with examples of the shape at the maximum and minimum of the first two canonical axes. If subfamilies are more or less separate from others, we coloured the max/min shape of the wing accordingly. Grey wing outlines represent the general shape if several subfamilies overlap. The outlines on the wing illustrations are interpretative and oriented on the specimen that has the minimum/maximum CV score on the respective axis. (TIF) [file pone.0275570.s001.tif]

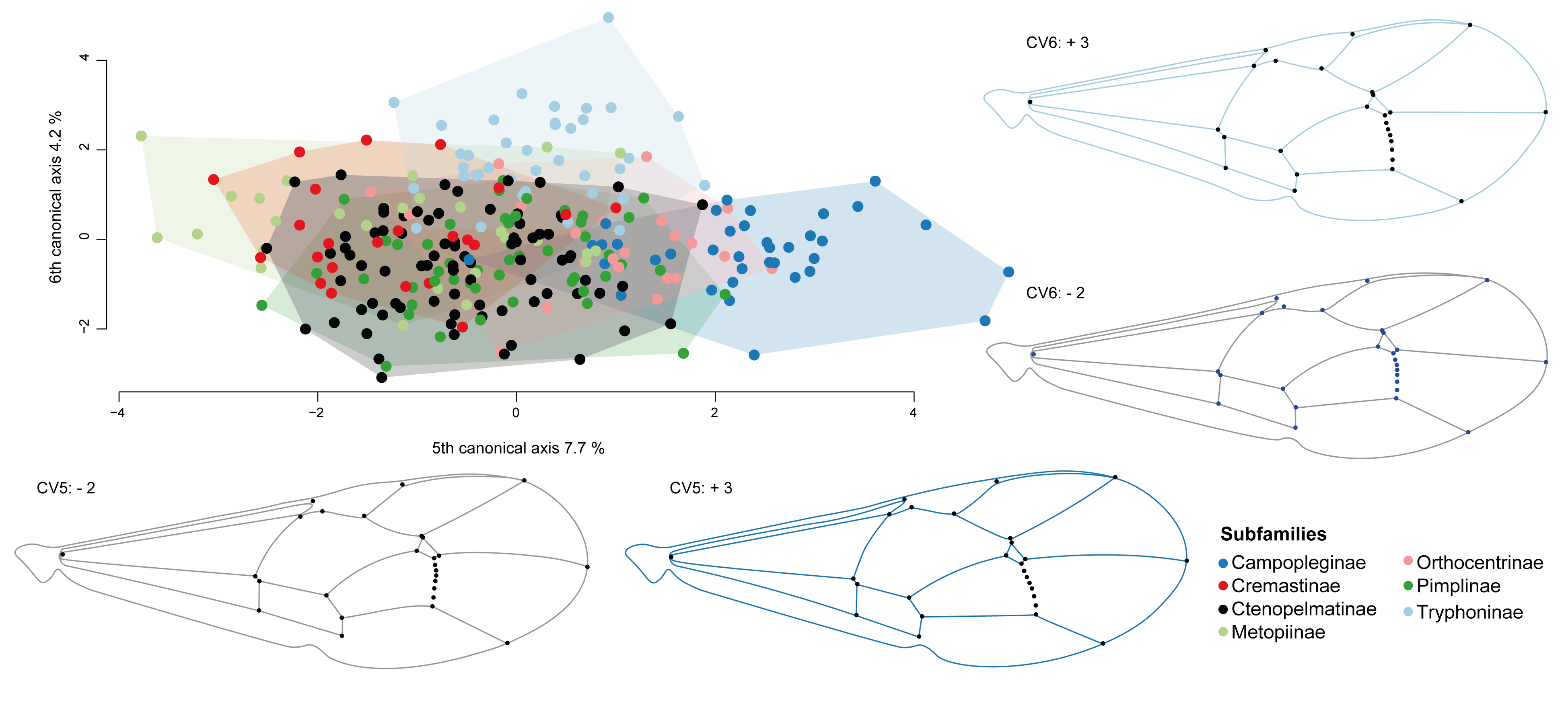

Supplement: S2 Fig — The fore wing shown in CV5 + 3 represents a Campopleginae fore wing, the fore wing in CV6 +3 represents a Tryphoninae fore wing. The other two greyish fore wing shapes represent the most common shape, where most species cluster in the plot. (TIF) [file pone.0275570.s002.tif]
